# Spatial transcriptomics of urothelial carcinoma with basal/squamous differentiation identifies Galectin-7 as a specific marker of squamous lineage commitment

**DOI:** 10.1007/s12672-026-04927-z

**Published:** 2026-03-31

**Authors:** Shunsuke Haga, Ryuta Watanabe, Mie Kurata, Noriyoshi Miura, Shuang Liu, Tadahiko Kikugawa, Riko Kitazawa, Masaki Mogi, Takashi Saika

**Affiliations:** 1 Department of Urology, Ehime University Graduate School of Medicine, Ehime University Hospital Shitsukawa, Toon, Ehime 7910295 Japan; 2 Department of Pharmacology, Ehime University Graduate School of Medicine, Toon, Japan; 3 Department of Analytical Pathology, Ehime University Graduate School of Medicine, Toon, Japan; 4 Department of Pathology, Ehime University Proteo-Science Center, Toon, Japan; 5 Division of Diagnostic Pathology, Ehime University Hospital, Toon, Japan

**Keywords:** Urothelial carcinoma, Basal/Squamous (Ba/Sq) differentiation, Galectin-7, Spatial transcriptomics, p63, NGFR

## Abstract

Urothelial carcinoma (UC) with basal/squamous (Ba/Sq) differentiation is an aggressive subtype associated with chemoresistance and poor prognosis. However, reliable biomarkers for its diagnosis remain limited, and current evaluation largely relies on morphology and p63 immunostaining, which lacks specificity. Using high-resolution spatial transcriptomics, we characterized the molecular continuum underlying Ba/Sq differentiation within UC and validated the findings by immunohistochemistry (IHC) in independent Ba/Sq UC and pure UC cohorts. Functional assays were performed in squamous carcinoma cell lines to assess the role of Galectin-7, a transcriptional target of p63, in cellular proliferation. Spatial analysis revealed a stromal–epithelial signaling axis driving Ba/Sq differentiation within UC, with NGFR-positive basal-like cells inducing Galectin-7 expression during early squamous commitment. Galectin-7 was absent in UC regions but strongly expressed in Ba/Sq-differentiated tumor nests. Its knockdown suppressed squamous carcinoma cell proliferation, supporting a functional role in tumor survival. Immunohistochemical analysis confirmed markedly higher specificity of Galectin-7 (92.2%) compared with p63 (12.5%), while maintaining high sensitivity (89.3% vs. 100%). Galectin-7 positivity was also associated with poorer progression-free survival, indicating its dual diagnostic and prognostic value. This study proposes a molecular and spatial framework for Ba/Sq differentiation within UC and identifies Galectin-7 as a specific diagnostic and prognostic marker, offering potential as a therapeutic target in Ba/Sq UC.

**Supplementary Information:**

The online version contains supplementary material available at 10.1007/s12672-026-04927-z.

## Introduction

Urothelial carcinoma (UC) is the most common malignancy of the urinary tract worldwide and poses a significant public health burden [1]. It is recognized as a major clinical challenge in the recent WHO Classification of Urinary and Male Genital Tumors (5th edition, 2022) [2] and reflected in current EAU and NCCN guidelines [3, 4]. Among its subtypes, muscle-invasive bladder cancer (MIBC) is particularly aggressive, often resistant to chemotherapy, and associated with poor prognosis [1].

Recent transcriptome-based studies have led to a comprehensive molecular classification of MIBC, stratifying tumors into luminal, stroma-rich, basal/squamous (Ba/Sq), and neuroendocrine-like categories [5, 6]. In this study, the term “basal/squamous (Ba/Sq) urothelial carcinoma” is used to refer to urothelial carcinomas exhibiting histologic squamous differentiation, consistent with the Ba/Sq molecular subtype described in prior classification studies. Prior work has also emphasized the clinical relevance of intrinsic basal and luminal subtypes in shaping bladder cancer biology and therapeutic responses [7]. More recently, single-cell and spatial transcriptomic analyses have revealed intratumoral subtype heterogeneity and dynamic plasticity in UC [8, 9], highlighting a biological continuum between urothelial and squamous phenotypes.

Among these molecular subtypes, Ba/Sq tumors are characterized by squamous differentiation accompanied by fibroblast and immune cell infiltration. Representing approximately 30–35% of MIBC, Ba/Sq tumors are consistently linked to worse clinical outcomes and divergent therapeutic responses [10–12]. Thus, their accurate identification is of substantial clinical importance.

Currently, diagnosis of Ba/Sq differentiation relies heavily on morphological evaluation, with few objective molecular markers available. The squamous marker p63 is commonly used; however, its expression is not specific, as it is also present in urothelial-type UC. Consequently, p63 lacks adequate discriminatory power for the early detection of Ba/Sq differentiation [13–15], reinforcing the need for more specific biomarkers.

Galectin-7 (encoded by *LGALS7*) is a β-galactoside-binding lectin family involved in apoptosis, cell adhesion, and inflammatory signaling [16]. It is highly expressed in normal stratified epithelia, and strong Galectin-7 expression has also been observed in benign squamous epithelial lesions such as cholesteatoma [17, 18]. Notably, Galectin-7 has been shown to regulate p63 expression through the JNK–miR-203 pathway in keratinocytes [19], suggesting a feedback loop rather than a linear downstream relationship between Galectin-7 and p63. However, the role of Galectin-7 in malignant squamous cell carcinomas, particularly in the Ba/Sq subtype of UC, remains largely uncharacterized.

In this study, we employed high-resolution spatial transcriptomics [20] to delineate the transcriptional continuum underlying Ba/Sq differentiation within UC. Within this framework, we investigated Galectin-7 in the context of the p63 regulatory axis and evaluated its utility as a marker of early squamous commitment. We further validated its diagnostic performance by immunohistochemistry, directly comparing Galectin-7 with p63 in Ba/Sq-differentiated UC. Our findings suggest that Galectin-7 is a highly specific immunohistochemical marker of Ba/Sq differentiation, providing a molecularly grounded framework for improved diagnosis and potential prognostic stratification.

## Results

### Case presentation and clustering

**Fig. 1:**
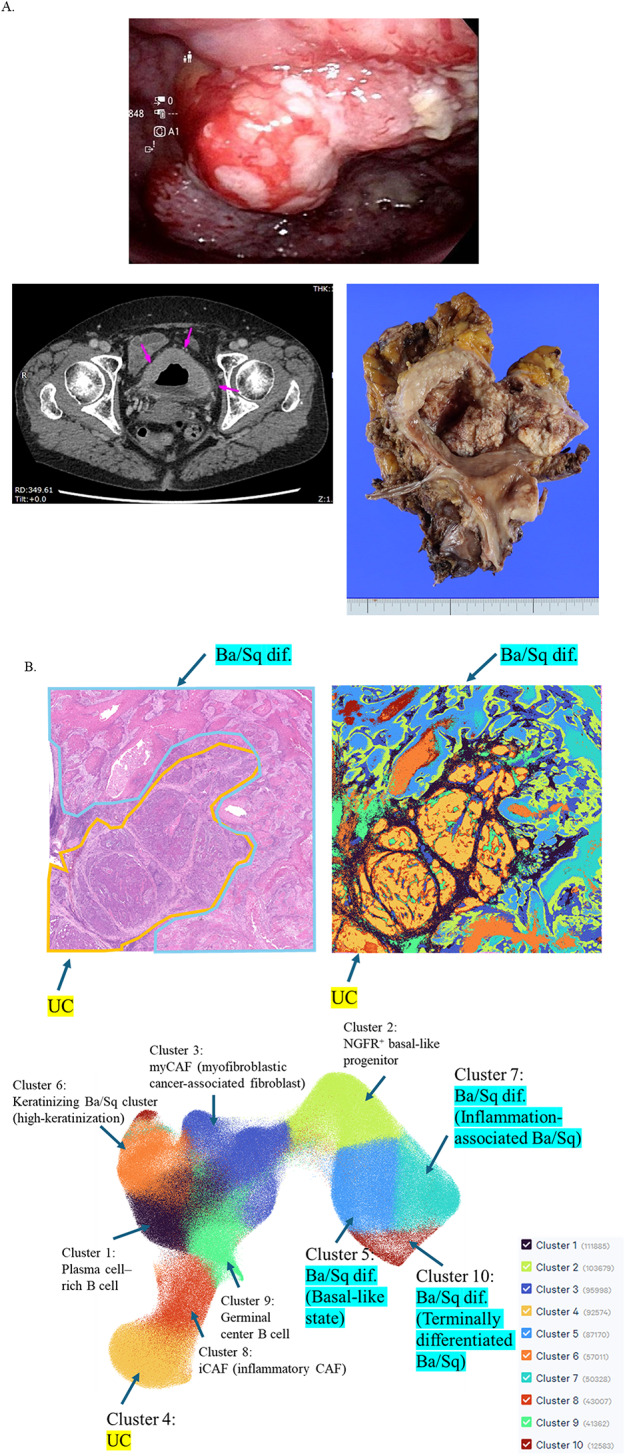

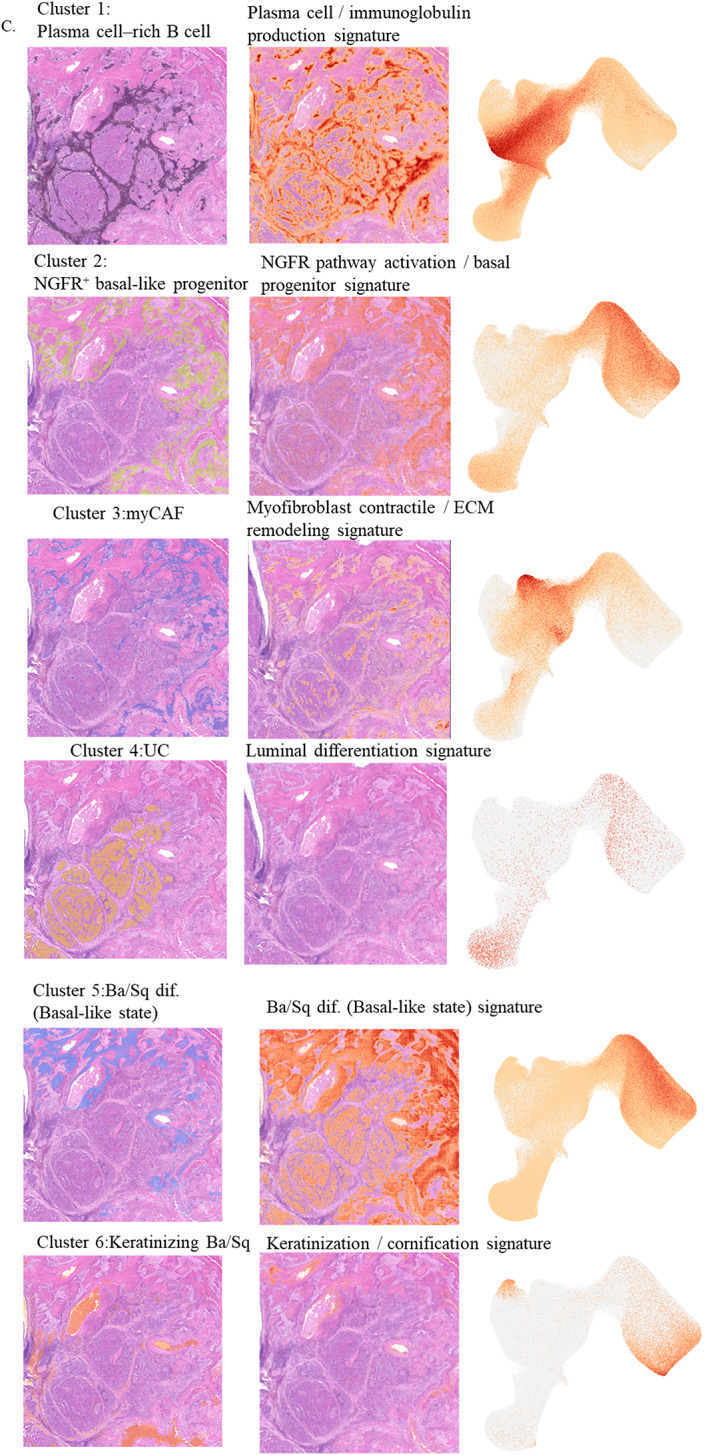

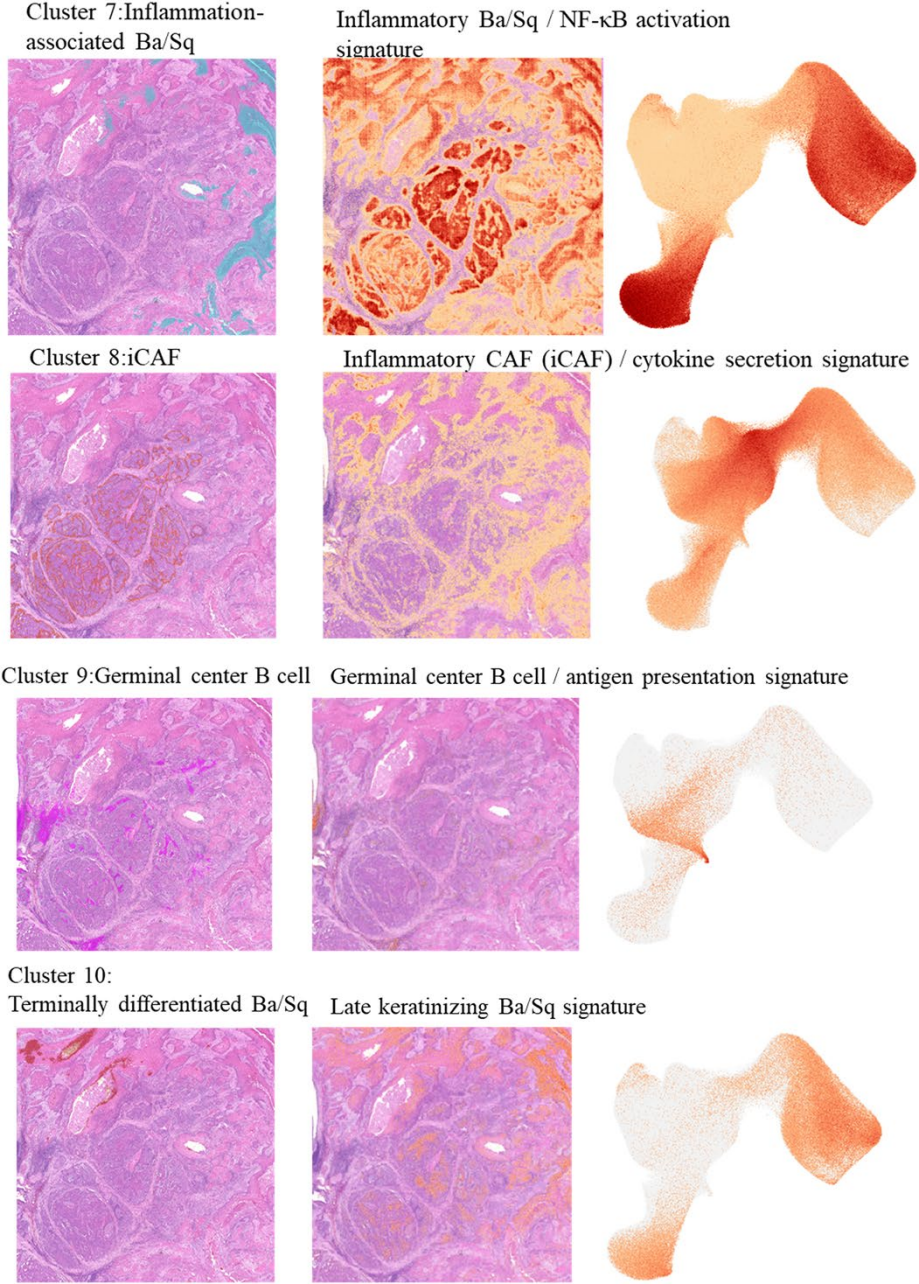
Case presentation and spatial clustering of urothelial carcinoma with Ba/Sq differentiation. **A** Representative cystoscopic, radiologic, and macroscopic findings of bladder carcinoma with Ba/Sq differentiation. Cystoscopy shows papillary to nodular, solid tumors on the anterior, left lateral, and posterior bladder walls (upper panel). Contrast-enhanced CT demonstrates irregular thickening of the anterior to left lateral wall (lower left). Macroscopic view of the radical cystectomy specimen reveals a solid tumor involving the anterior to left lateral wall (lower right). **B** Spatial transcriptomic clustering of the cystectomy section using Visium HD, revealing 10 transcriptionally distinct clusters. **C** Spatial distribution of each cluster along with expression signatures of representative marker genes

We analyzed a prototypical case of bladder carcinoma with squamous differentiation. The patient was a 71-year-old woman who presented with gross hematuria. Contrast-enhanced CT revealed irregular thickening of the anterior to left lateral bladder wall, without involvement of the upper urinary tract or evidence of distant metastasis (Fig. 1A). Cystoscopy revealed papillary to nodular lesions in the same regions (Fig. 1A). Transurethral resection of the bladder tumor (TUR-Bt) confirmed a diagnosis of squamous cell carcinoma with at least muscularis propria invasion (pT2). Given the limited efficacy of neoadjuvant chemotherapy for pure SCC, the patient underwent radical cystectomy with ileal conduit diversion and pelvic lymph node dissection (Fig. 1A).

Final pathology revealed invasive urothelial carcinoma with squamous differentiation (pT3bN0M0), with negative surgical margins (INFb, LV0, V1, RM0). This case was considered representative of UC with squamous differentiation and was therefore well selected for spatial transcriptomic analysis.

A representative section from the cystectomy specimen, encompassing both UC and Ba/Sq-differentiated regions, was subjected to high-resolution spatial transcriptomic profiling using Visium HD. The analysis identified 10 distinct transcriptional clusters across the tumor section (Fig. 1B). Each cluster was annotated based on the expression profile of canonical marker genes, as detailed in the Methods section and Table 1.

We next visualized the spatial distribution of each cluster and the expression patterns of representative marker genes (Fig. 1C). Cluster 4 corresponded to UC, marked by high expression of luminal UC markers such as GATA3, UPK family genes, and KRT20. Ba/Sq-differentiated regions were classified into four clusters (5, 6, 7, and 10). Cluster 5, predominantly located at the UC–Ba/Sq interface, showed elevated expression of TP63, KRT5/14, and DSG3, consistent with a basal-like Ba/Sq phenotype. Cluster 6 expressed keratinization markers including IVL, SPRR family genes, and LOR, consistent with keratinizing Ba/Sq differentiation. Cluster 7 retained epithelial/Ba/Sq-associated markers while showing upregulation of inflammatory and neutrophil-associated genes such as S100A8/A9, IL1B, and CXCL8, defining an inflammation-associated Ba/Sq subtype enriched at the tumor periphery. Cluster 10 was marked by strong expression of terminal differentiation genes including KLK7, KLK5, and LCE family members, indicative of late keratinizing Ba/Sq differentiation.

Within the tumor stroma, two distinct fibroblast clusters were identified. Cluster 8, located along the UC border, showed elevated expression of ECM remodeling and TGF-β-associated genes such as COL1A1, DCN, FAP, and POSTN, consistent with an inflammatory cancer-associated fibroblast (iCAF) phenotype. In contrast, Cluster 3, predominantly found within Ba/Sq-differentiated tumor cores, expressed myofibroblastic and contractile markers such as ACTA2, TAGLN, and MYL9, consistent with myCAF characteristics. These spatially complementary distributions are consistent with a model in which distinct CAF populations may contribute to Ba/Sq differentiation within UC.

Immune cell clusters were also identified, including plasmacytoid B cells (Cluster 1) and germinal center B cells (Cluster 9), both enriched near CAF-dense regions. Notably, Cluster 2 was localized along the UC–Ba/Sq boundary and expressed high levels of NGFR, KRT14, ITGA6, and TP63, representing a basal-like progenitor population that may reflect a transitional state preceding squamous commitment.

### Activation of NGFR signaling at the UC–Ba/Sq interface driven by stromal cues

As described above, unsupervised clustering identified ten transcriptionally distinct clusters across the tumor section (Fig. 1B). We next focused on their functional annotation to better understand the biological programs associated with each cluster. Differential expression analysis identified the top 20 upregulated genes for each cluster, which were used for functional annotation (Supplementary Figure A). Pathway enrichment analyses further characterized the dominant signaling programs (Supplementary Figure B).

Clusters 8 and 3 corresponded to spatially segregated fibroblast programs. Cluster 8, forming a rim around the UC regions, exhibited an inflammatory CAF (iCAF)-like signature, characterized by high expression of *FAP*,* COL1A1*,* DCN*,* POSTN*,* FN1*, and *CXCL12*. Pathway enrichment indicated activation of extracellular matrix organization, TGF-β signaling, and chemokine-mediated signaling, suggesting the presence of a cytokine-rich stromal niche remodeling the tumor-stromal interface.

In contrast, Cluster 3 expanded within Ba/Sq-differentiated tumor nests and displayed a myofibroblastic CAF (myCAF)-like program, marked by elevated expression of ACTA2, TAGLN, and DES, with enrichment of smooth muscle contraction and myofibroblast activation pathways. These features support a contractile, matrix-remodeling phenotype that may stabilize Ba/Sq tumor architecture.

Two transcriptionally distinct B-cell populations were identified. Cluster 1 showed high expression of IGKC, IGHG1, IGHA1, MZB1, and IGLC1, consistent with a plasma cell–like phenotype enriched within stromal regions located between UC and Ba/Sq-differentiated regions. Pathway enrichment suggested active immunoglobulin production and a prominent humoral immune response. In contrast, Cluster 9 expressed MS4A1 and CD79A, consistent with a germinal center-like B-cell program, and was primarily located within UC regions.

The spatial proximity of Cluster 1 to the iCAF-rich Cluster 8 suggests potential B-cell–CAF interactions. In this context, cytokines secreted by B-cells, such as IL-6, TNF, and CXCL12, may influence fibroblast activation and promote a phenotypic shift toward myCAF under TGF-β–dominant microenvironment conditions.

**Fig. 2:**
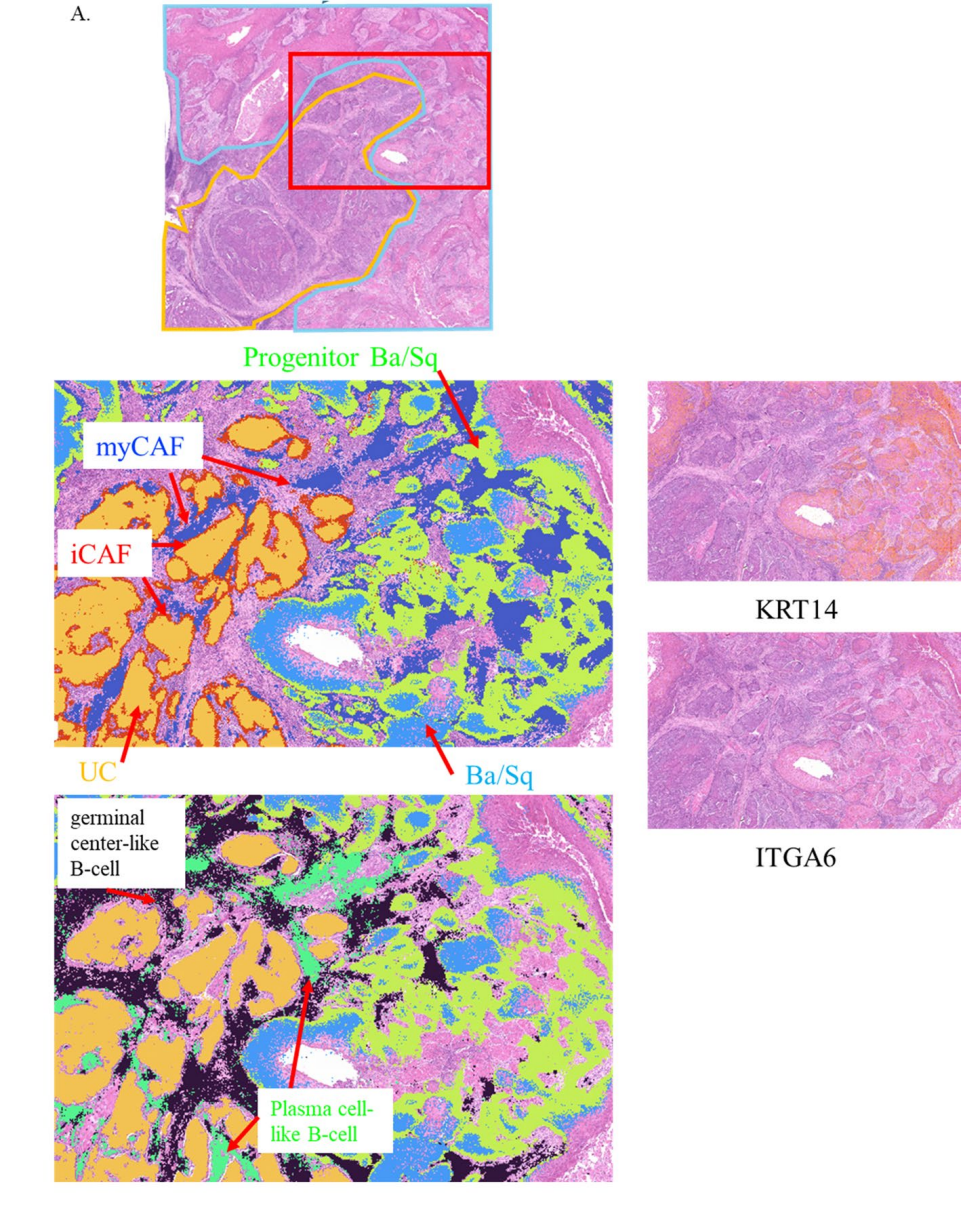

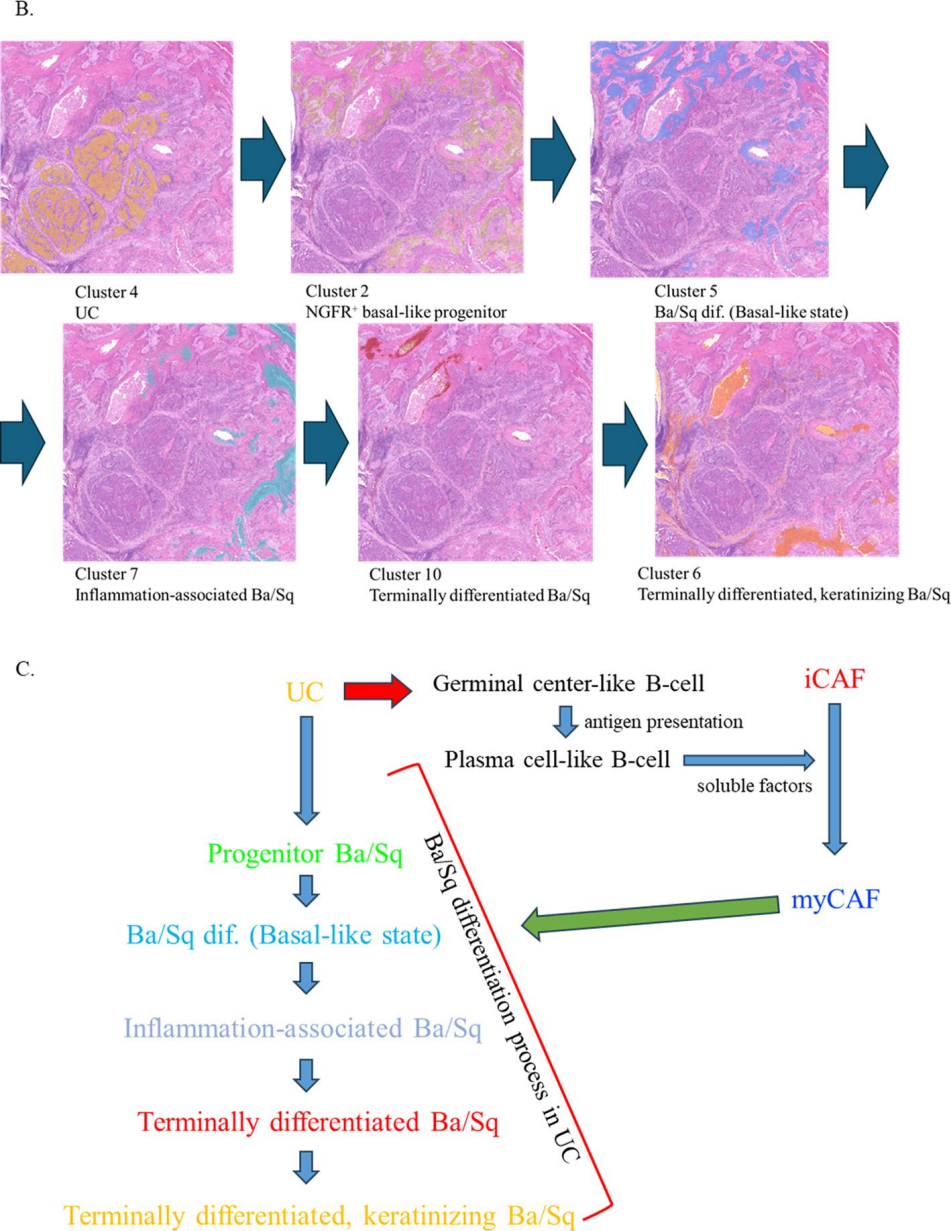
Spatial transcriptomic analysis of UC with Ba/Sq differentiation. **A** The H&E image from Fig. 1B is shown again, with a red box indicating the region subjected to spatial transcriptomic analysis in this figure. Feature plots of NGFR, KRT14, and ITGA6 expression highlight Cluster 2 at the UC–Ba/Sq interface within the boxed area. **B** Stepwise trajectory from fibroblast- and plasma cell–rich niches (Clusters 8 and 1), through NGFR/basal-marker–high transitional epithelium (Cluster 2), to basal-like Ba/Sq differentiation (Cluster 5) and keratinizing Ba/Sq differentiation (Clusters 6 and 10). **C** Schematic representation of Ba/Sq differentiation progression from early to late stages, with fibroblasts (iCAFs and myCAFs) and immune cells, including T cells, acting as intermediaries that remodel the tumor microenvironment and promote basal epithelial commitment toward squamous differentiation

Cluster 2 was defined by high NGFR expression, along with basal stem-like markers including KRT14 and ITGA6 (Fig. 2A). Pathway analysis revealed activation of neurotrophin signaling and p75NTR-related pathways, suggesting a role for this cluster as a transitional gate at the UC–Ba/Sq interface. Spatially, Cluster 2 localized adjacent to fibroblast- and plasma cell–rich regions, supporting a model in which paracrine stromal and immune-derived cues activate NGFR to initiate squamous differentiation in epithelial cells. Cluster 2 represents a transitional epithelial state marked by high NGFR expression, which may function as a signaling integrator for stromal and inflammatory cues associated with early squamous commitment.

Cluster 5 was enriched for basal-like markers characteristic of early Ba/Sq differentiation including TP63, KRT5, and DSG3, with pathway enrichment for epithelial cell differentiation and p63-regulated transcriptional programs. This cluster likely represents the basal Ba/Sq compartment that arises downstream of NGFR activation.

Clusters 6 and 10 exhibited terminal keratinocyte differentiation signatures. Cluster 6 expressed IVL, SPRR family genes, and LOR, while Cluster 10 was marked by high expression of LCE family genes and kallikreins. Pathway enrichment analysis revealed robust activation of keratinization, cornified envelope formation, and epidermal differentiation programs, consistent with progressive maturation toward keratinizing Ba/Sq phenotypes.

Collectively, these results are consistent with a stepwise transcriptional trajectory extending from stromal–immune–rich regions (Clusters 8 and 1), through an NGFR- and basal marker–high transitional epithelial state (Cluster 2), toward basal-like Ba/Sq differentiation (Cluster 5), and ultimately to terminally differentiated keratinizing Ba/Sq states (Clusters 6 and 10) (Fig. 2B). The spatial juxtaposition of iCAF-like Cluster 8, plasma cell–rich Cluster 1, and myCAF-like Cluster 3 further supports the existence of a coordinated stromal–immune–epithelial signaling axis that remodels the microenvironment, primes basal epithelial cells, and facilitates Ba/Sq differentiation within UC (Fig. 2C).

### LGALS7 is selectively expressed in early-stage Ba/Sq differentiation and declines with terminal differentiation

**Fig. 3:**
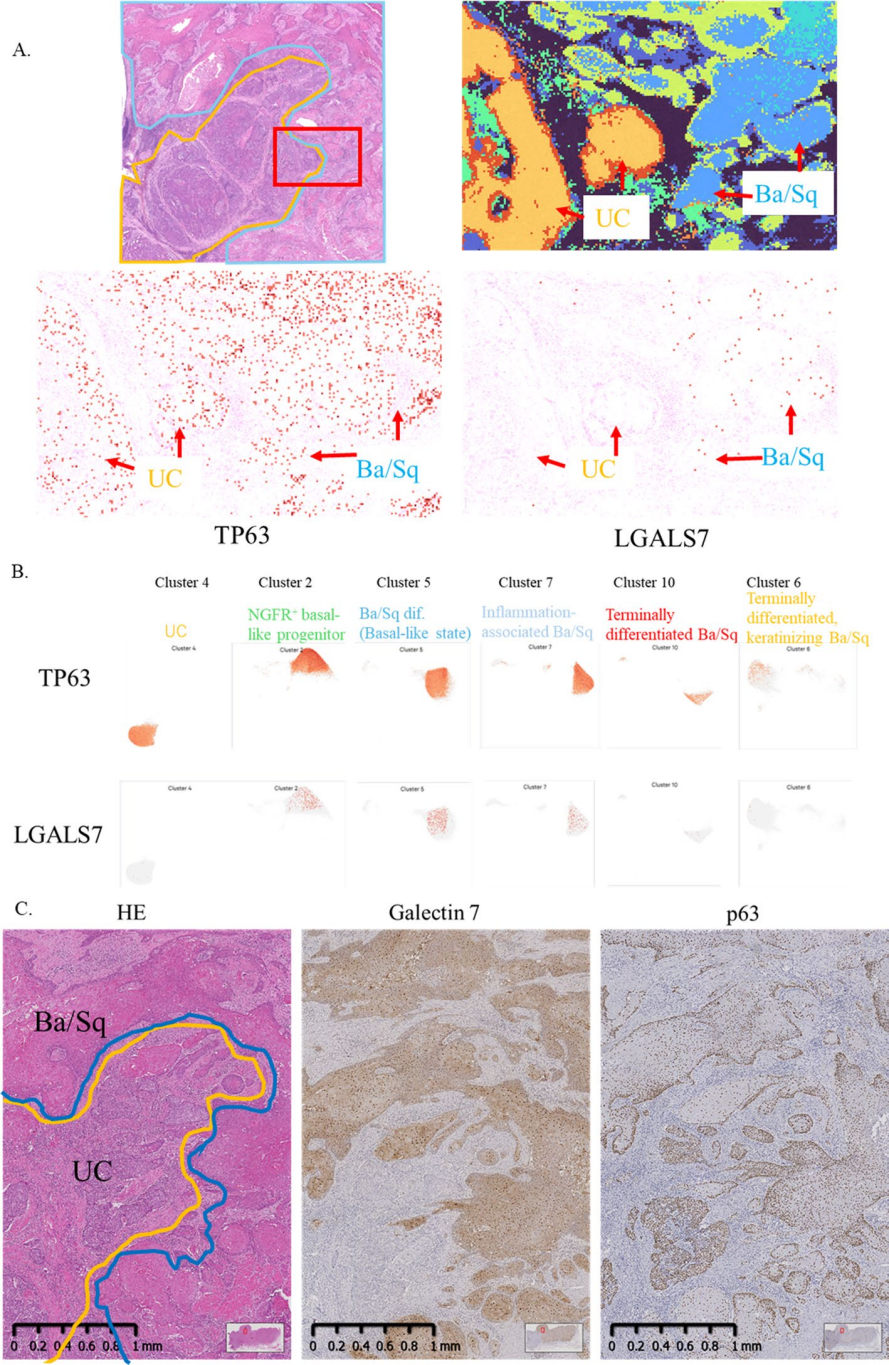

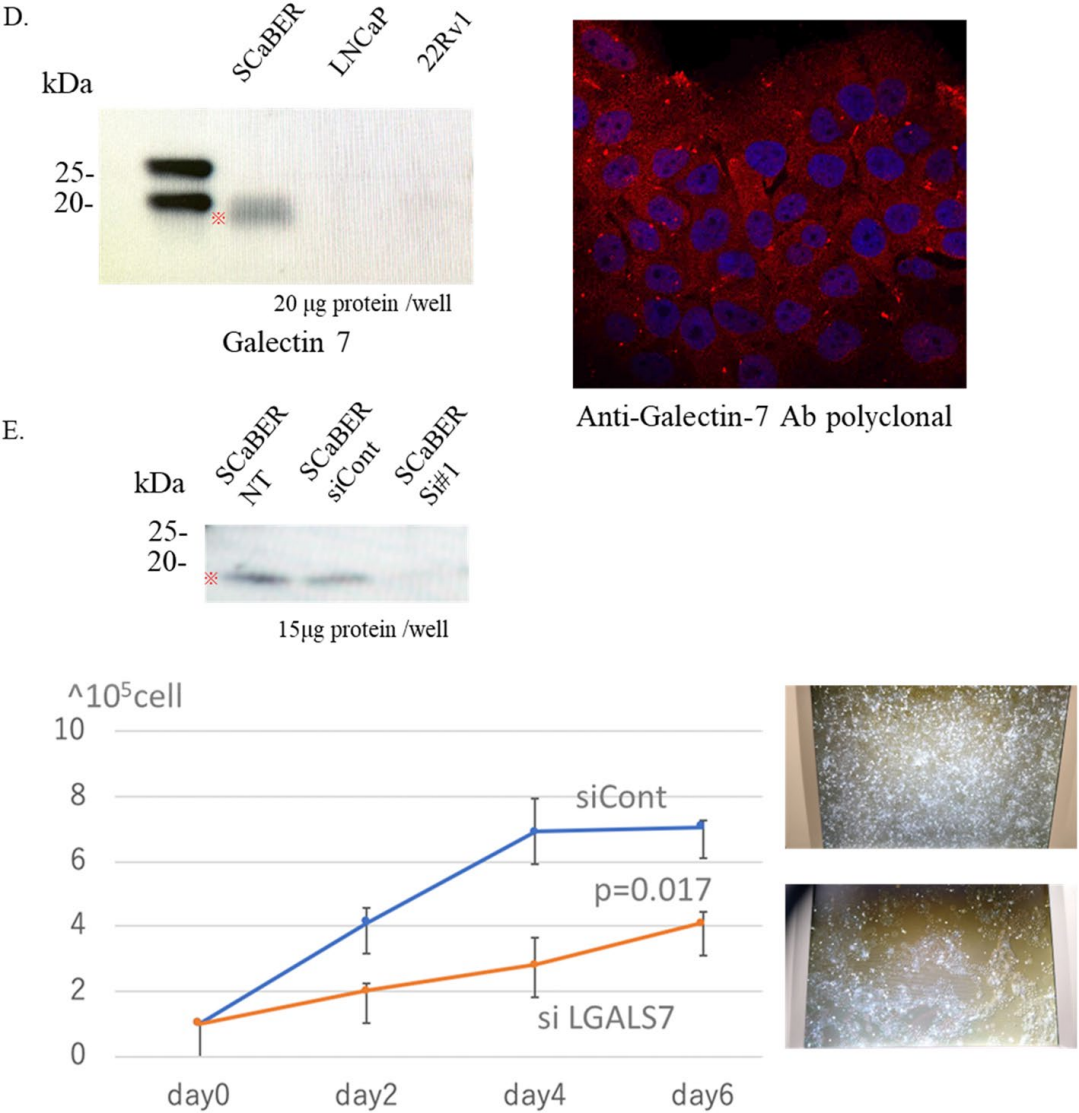
LGALS7 is selectively expressed in Ba/Sq differentiation and promotes squamous carcinoma cell proliferation. **A** The H&E image from Fig. 1B is shown again, with a red box indicating the region analyzed in this figure. Spatial transcriptomic maps display TP63 and LGALS7 expression patterns within the boxed region, corresponding to the UC–Ba/Sq interface. **B** Cluster-level expression of TP63 and LGALS7. C Immunohistochemistry (IHC) of the cystectomy specimen showing diffuse p63 positivity in both urothelial carcinoma (UC) and Ba/Sq-differentiated tumor components, and selective Galectin-7 expression restricted to Ba/Sq-differentiated tumor nests. Scale bars, 1 mm. **D** Western blot showing Galectin-7 expression across cancer cell lines (left), and immunofluorescence (IF) of SCaBER cells showing cytoplasmic localization (right). **E** Proliferation assay of SCaBER cells following LGALS7 knockdown

To identify a more specific marker distinguishing Ba/Sq differentiation from UC, we first examined the expression of TP63, a widely used diagnostic marker of squamous differentiation. Spatial transcriptomic analysis revealed diffuse TP63 expression across both Ba/Sq-differentiated and UC regions, limiting its diagnostic specificity (Fig. 3A, left). We next investigated LGALS7 (Galectin-7), a known transcriptional target of p63. In contrast to TP63, LGALS7 expression was strictly confined to Ba/Sq-differentiated regions and absent from UC, with the highest expression observed in early Ba/Sq-differentiated tumor nests and a progressive decline toward terminally keratinized regions (Fig. 3A, right).

Cluster-level analysis supported this pattern: TP63 was broadly expressed across multiple clusters, including UC, whereas LGALS7 was selectively enriched in the basal-like cluster characteristic of early Ba/Sq differentiation (Cluster 5) and partially in NGFR⁺ transitional epithelium (Cluster 2), but was nearly absent in terminally differentiated Ba/Sq clusters (Clusters 6/10) (Fig. 3B). These findings suggest that LGALS7 marks the early stages of squamous differentiation and may serve as a more specific biomarker for Ba/Sq differentiation than TP63.

To validate the spatial transcriptomic findings at the protein level, immunohistochemistry for p63 and Galectin-7 was performed on the same cystectomy specimen used for Visium HD analysis. Consistent with the transcriptomic data, p63 was broadly expressed in both UC and Ba/Sq-differentiated tumor components, whereas Galectin-7 expression was restricted to Ba/Sq-differentiated areas, confirming its selective association with squamous differentiation (Fig. 3C). This concordance between mRNA and protein level expression supports the specificity of Galectin-7 as a marker of squamous differentiation.

We further assessed Galectin-7 protein expression across cancer cell lines using Western blotting. Strong expression was detected in the squamous carcinoma cell line SCaBER, while Galectin-7 was undetectable in the prostate cancer cell lines LNCaP and 22Rv1 (Fig. 3D, left). Immunofluorescence confirmed cytoplasmic localization of Galectin-7 in SCaBER cells (Fig. 3D, right). Furthermore, siRNA-mediated knockdown of LGALS7 in SCaBER cells significantly reduced proliferation compared with control cells, as measured by direct cell counting (*p* = 0.017) (Fig. 3E). Collectively, these findings suggest that Galectin-7 not only serves as a diagnostic marker of squamous differentiation but may also functionally contribute to squamous carcinoma cell survival and proliferation.

### Diagnostic utility of Galectin-7 in identifying Ba/Sq differentiation

**Fig. 4:**
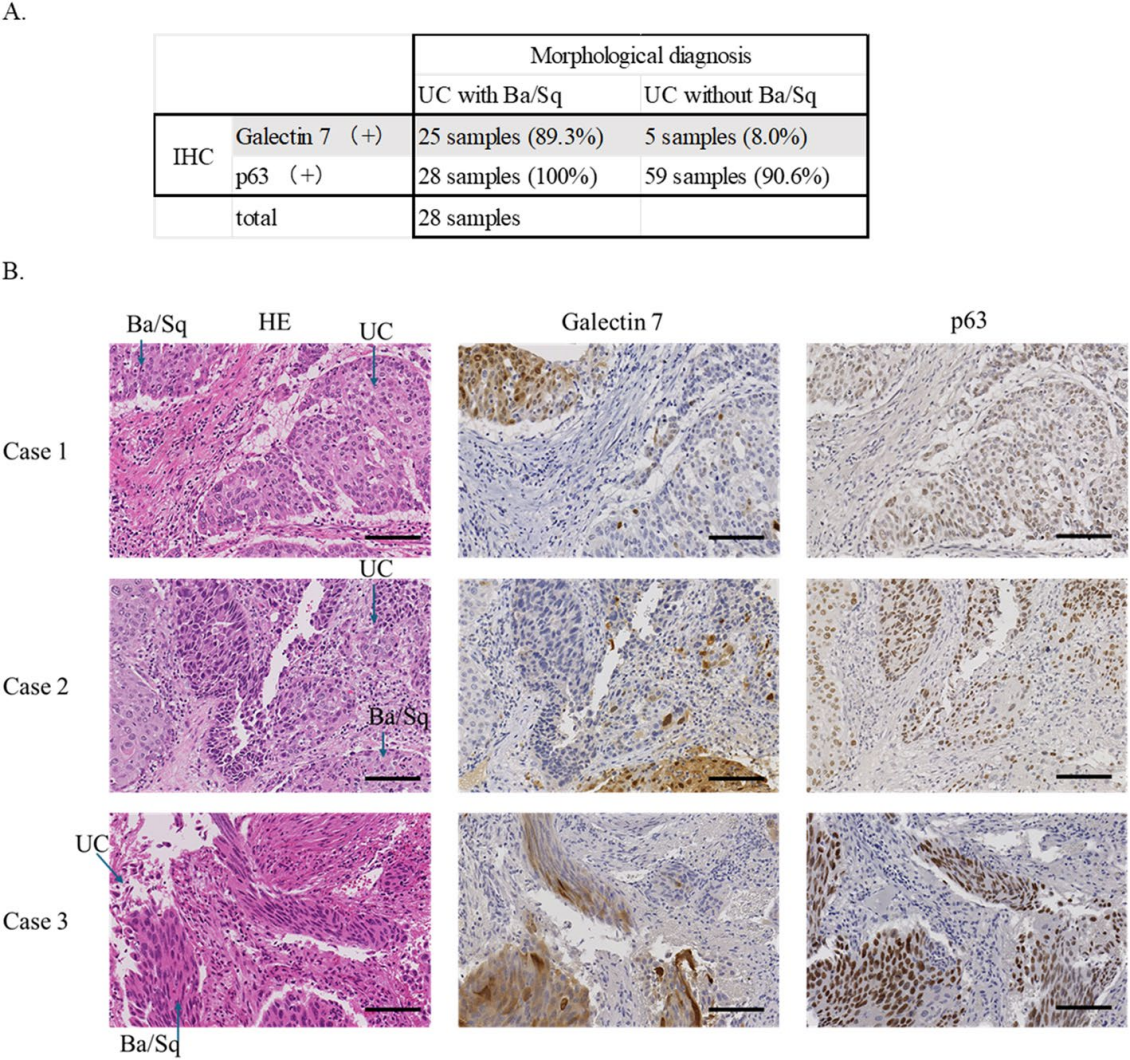

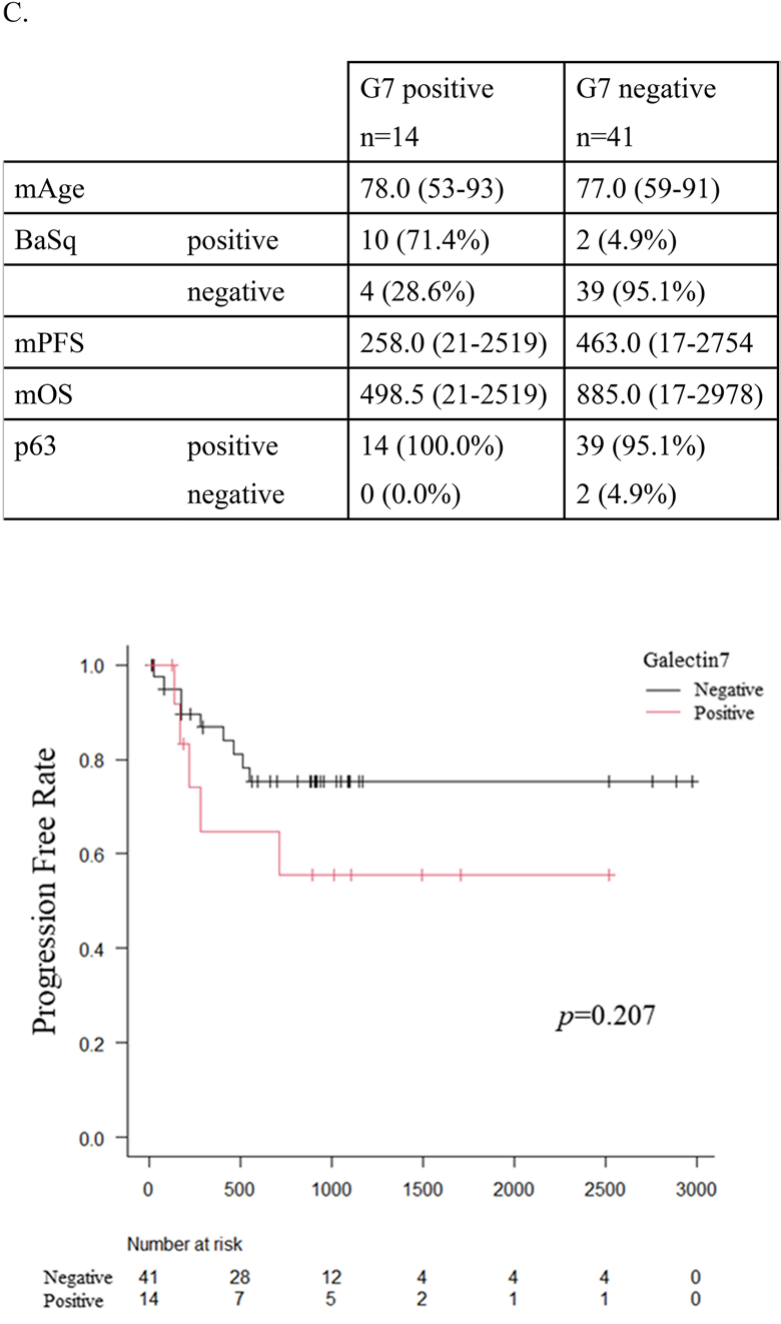
Diagnostic and prognostic value of Galectin-7 in urothelial carcinoma. **A** Immunohistochemistry (IHC) for p63 and Galectin-7 in UC with Ba/Sq features (*n* = 28) and UC without Ba/Sq (*n* = 64). **B** Immunohistochemistry (IHC) of three representative UC with Ba/Sq differentiation cases showing diffuse p63 positivity in both urothelial carcinoma (UC) and Ba/Sq-differentiated tumor components, and selective Galectin-7 expression restricted to Ba/Sq-differentiated tumor nests. Scale bars, 100 μm. **C** Summary of key clinicopathological parameters associated with Galectin-7 (G7) expression. Detailed clinicopathological data are provided in Supplementary Table 1. Kaplan–Meier analysis of progression-free survival stratified by Galectin-7 expression

To assess the diagnostic performance of Galectin-7 in identifying squamous differentiation within urothelial carcinoma (UC), we performed immunohistochemical (IHC) analysis on 28 cases of Ba/Sq UC and 64 cases of pure UC. Both p63 and Galectin-7 were evaluated in parallel, using histopathological diagnosis as the reference standard (Fig. 4A).

Galectin-7 demonstrated a sensitivity of 89.3% and a specificity of 92.2%. In contrast, p63 showed 100% sensitivity but only 7.8% specificity, indicating that although p63 reliably detects squamous differentiation, it lacks specificity due to its expression in UC. Galectin-7, by comparison, offered a more balanced and diagnostically informative profile.

Representative histological images of three independent UC with Ba/Sq differentiation cases are shown in Fig. 4B. Serial sections stained for p63 and Galectin-7 demonstrated consistent findings across all cases: while p63 was diffusely expressed in both UC and Ba/Sq-differentiated areas, Galectin-7 expression was confined to Ba/Sq-differentiated tumor nests. These results further support the specificity of Galectin-7 as a marker of squamous differentiation in urothelial carcinoma.

We next examined the prognostic relevance of Galectin-7 expression (Fig. 4C). Kaplan–Meier analysis was performed in patients with ≥T2 tumors who had not received neoadjuvant chemotherapy or BCG therapy. Among these, 14 cases were Galectin-7–positive and 41 were Galectin-7–negative. Although the difference did not reach statistical significance (log-rank test, *p* = 0.207), Galectin-7–positive cases tended to show shorter progression-free survival (PFS) compared with negative cases, suggesting a possible prognostic trend that may become significant with larger sample sizes. Notably, even within histologically defined Ba/Sq cases, Galectin-7 expression further stratified prognosis beyond morphology alone. To further examine whether this association was influenced by tumor stage, additional subgroup analyses were performed stratified by pathological T stage, N stage, and overall clinical stage (Supplementary Figure S3). Within individual strata, Galectin-7 expression did not demonstrate statistically significant differences in PFS or OS. However, no consistent inverse trends were observed across strata, suggesting that the prognostic effect of Galectin-7 may not be solely attributable to TNM stage differences. These subgroup analyses were limited by small sample sizes in each category, resulting in wide confidence intervals and reduced statistical power.

These findings indicate that Galectin-7 serves as a diagnostic marker for Ba/Sq differentiation and may also have a prognostic value in patients with UC.

## Discussion

However, accurate identification of Ba/Sq differentiation in routine clinical practice remains challenging. Current diagnosis primarily relies on morphological assessment using H&E staining, often supplemented with immunohistochemical markers such as p63. Although p63 has high sensitivity, its diagnostic specificity is limited due to its broad expression in both UC and Ba/Sq-differentiated tumor components, making it suboptimal as a Ba/Sq-specific marker. Therefore, there is a clear need to identify more specific biomarkers that can enhance diagnostic accuracy and inform therapeutic decision-making.

To address this unmet need, we employed high-resolution Visium HD spatial transcriptomics to reconstruct the molecular and spatial continuum of the Ba/Sq differentiation process within UC (Fig. 2D and E). This analysis revealed a sequential transcriptional trajectory: (i) CAF-rich stroma enriched in TGF-β and extracellular matrix programs; (ii) NGFR-high transitional epithelium at the UC–stroma interface; (iii) basal-like Ba/Sq-differentiated tumor nests expressing TP63 and its downstream effector LGALS7 (Galectin-7); and (iv) terminally keratinized Ba/Sq-differentiated tumor cores dominated by epidermal differentiation complex (EDC) genes. These findings support a model in which stromal–epithelial crosstalk may contribute to squamous lineage commitment through p63-associated transcriptional programs and progressive keratinization. This stepwise molecular framework offers mechanistic insight into Ba/Sq differentiation within UC and forms the basis for the analyses presented below.

Given the association of Ba/Sq features with chemoresistance, poor survival, and limited therapeutic options [1, 21], early and accurate detection of squamous differentiation within UC is of high clinical significance. However, current diagnostic approaches remain reliant on histomorphological criteria, and no robust molecular markers have been established to date [22].

To investigate potential microenvironmental cues driving this transition, we analyzed the UC–Ba/Sq interface. At this interface, cancer-associated fibroblasts (CAFs) formed a transcriptionally distinct cluster enriched in matrisome-related genes (COL1A1, FN1, POSTN, DCN) and TGF-β–responsive transcripts such as FAP and CXCL12. Pathway analysis confirmed enrichment in extracellular matrix organization, NABA matrisome, and TGF-β signaling, indicating a stiffened, signaling-active microenvironment. These findings are consistent with previous studies in breast and pancreatic cancers, where CAF-mediated ECM remodeling and paracrine signaling have been shown to promote epithelial plasticity and lineage switching [23, 24].

Recent studies have highlighted the critical role of the tumor immune microenvironment and stromal–epithelial interactions in driving lineage plasticity and squamous differentiation in urothelial carcinoma and other epithelial malignancies [25–27].

Spatially, these CAF-rich regions were contiguous with UC epithelium and directly abutted NGFR-high clusters, indicating a potential niche for stromal–epithelial interactions. The enrichment of TGF-β signaling in this region is particularly relevant, given its established role in modulating the tumor microenvironment, promoting squamous differentiation, and contributing to therapy resistance [24, 28]. Our data support a model in which CAFs create an initiating niche that primes adjacent UC epithelium toward basal reprogramming and the onset of squamous lineage differentiation.

The transitional epithelial cluster adjacent to the CAF-rich stroma was characterized by high expression of NGFR (p75^NTR^), along with basal/stem markers such as KRT14 and ITGA6, and reduced expression of luminal UC markers including GATA3 and UPK genes. NGFR is a well-recognized marker of basal progenitor populations in stratified epithelia, including oral keratinocytes and airway epithelium [29, 30]. Beyond serving as a marker, NGFR also acts as a signaling integrator of neurotrophin, TNF-family, and ECM-derived cues, activating downstream JNK, NF-κB, or apoptotic pathways in a context-dependent manner [31].

Previous reports in head and neck squamous cell carcinoma have described transcriptionally diverse basal-like cells with high plasticity and therapy resistance[32]. In our dataset, NGFR-high UC epithelium localized adjacent to TGF-β–active CAFs and inflammatory niches (IL1B⁺, TNF⁺), suggesting that these epithelial cells are “poised responders” to stromal signals and may function as early gatekeepers of squamous lineage commitment.

Within the basal-like cluster characteristic of early Ba/Sq differentiation, TP63 and keratinocyte-associated genes (KRT5, DSG3, SFN) were highly upregulated alongside LGALS7 (Galectin-7). Galectin-7 is a direct transcriptional target of p63, with prior studies demonstrating p63 binding to the LGALS7 promoter and induction of its transcription during keratinocyte differentiation [16]. Our data showed that Galectin-7 expression peaked at the early Ba/Sq differentiation stage and declined with terminal keratinization, supporting its role as an early “switch-on” marker of squamous differentiation. Unlike p63, which is broadly expressed across both UC and Ba/Sq-differentiated tumor regions, Galectin-7 was absent in UC regions and confined to Ba/Sq-differentiated tumor nests, indicating higher diagnostic specificity.

Functionally, Galectin-7 regulates keratinocyte apoptosis, migration, and tissue homeostasis via JNK and p53 pathways [16], and has been shown to enhance metastatic potential in breast cancer models [33]. These functions provide a mechanistic rationale for selecting Galectin-7 as the focus of our study. It is not merely a correlative marker but a p63-driven effector that participates in the differentiation process. Furthermore, our knockdown experiments in SCaBER cells demonstrated that Galectin-7 promotes tumor cell proliferation. This finding aligns with prior reports implicating Galectin-7 in tumor progression and metastasis across multiple cancer types [33, 34]. These data suggest that Galectin-7 may serve not only as a marker of squamous differentiation but also as a potential therapeutic target in Ba/Sq UC.

Although Galectin-7 was evaluated exclusively by immunohistochemistry in the present study, its biological properties raise the possibility that it could serve as a non-invasive biomarker in the future. Recent advances in urinary molecular diagnostics, including DNA methylation–based assays, have demonstrated that non-invasive biomarkers can complement conventional urinary cytology for the detection and characterization of urothelial carcinoma [35, 36]. In this context, detection of Galectin-7 in urine or blood may potentially reflect underlying basal/squamous differentiation within UC, although this possibility remains to be formally investigated.

At present, whether Galectin-7 can be reliably detected in urine or plasma, and whether such measurements would provide sufficient sensitivity and specificity for clinical application, remains unknown. Nevertheless, given the well-recognized limitations and interobserver variability of morphology-based assessment in variant histologies [37], incorporation of Galectin-7 into liquid biopsy–based approaches may represent a complementary strategy to existing diagnostic frameworks, such as the Paris System for urinary cytology, particularly in cases with equivocal morphology or early Ba/Sq differentiation.

Beyond histopathological evaluation, a growing body of evidence indicates that molecular biomarkers, including epigenetic and transcriptomic signatures, can provide prognostic information beyond conventional morphology in urothelial carcinoma [38]. In parallel, PD-L1–related biomarkers, assessed either by immunohistochemistry, epigenetic regulation, or integrative multi-omics approaches, have been shown to carry both predictive and prognostic significance [39–41]. Importantly, the potential role of Galectin-7 differs conceptually from these established biomarkers: rather than directly informing immunotherapy eligibility, Galectin-7 may function as a lineage- and differentiation-associated marker that adds pathological context and may aid patient stratification prior to therapeutic decision-making.

In addition to CAF activation at the UC–Ba/Sq interface, we identified distinct CAF subtypes: inflammatory CAFs (iCAFs, CXCL12⁺/IL6⁺/TGF-β–active) localized at the boundary, and myofibroblastic CAFs (myCAFs, αSMAhigh, enriched for contractile genes) located deeper within Ba/Sq-differentiated tumor nests. This spatial arrangement supports the concept of iCAF-to-myCAF plasticity, consistent with observations in pancreatic cancer, where CAFs adaptively transition under sustained TGF-β signaling and ECM stiffening [42, 43]. Functionally, iCAFs may facilitate squamous initiation in adjacent UC epithelium, whereas myCAFs may reinforce terminal keratinization and contribute to stromal rigidity.

We also detected two transcriptionally distinct B-cell clusters: germinal center-like B cells located within the UC compartment, and plasma cell-like populations situated in the stroma between UC and Ba/Sq-differentiated regions. The latter population was spatially juxtaposed with iCAFs, raising the possibility of B cell–CAF crosstalk mediated by factors such as CXCL12, IL-6, and TNF. Previous studies have implicated B cells in modulating fibroblast phenotypes and promoting tumor-supportive inflammation [44, 45]. Although speculative, our data suggest that immune–stromal crosstalk may contribute an additional regulatory layer to the Ba/Sq differentiation process within UC.

Overall, these findings support an integrated model wherein stromal signals—including TGF-β, ECM stiffness, and inflammatory cytokines—activate NGFR-positive basal-like progenitor cells. This activation triggers p63-driven transcriptional reprogramming and induction of Galectin-7, leading to squamous differentiation and culminating in terminal keratinization. The observed heterogeneity in CAF populations, immune–stromal interactions, and lineage-specific transcriptional programs converge to construct a biologically plausible and spatially coherent framework for Ba/Sq differentiation within UC.

Although these findings were derived from a single representative case, the stepwise model integrates multiple lines of spatial and molecular evidence and provides a foundation for validation in larger cohorts. Beyond its mechanistic contributions, this model identifies novel molecular checkpoints—particularly NGFR and Galectin-7—that could serve as diagnostic or therapeutic targets in Ba/Sq UC.

To validate our transcriptomic findings, we performed immunohistochemistry on 28 Ba/Sq UC and 64 pure UC cases. Compared to p63, Galectin-7 demonstrated substantially higher specificity (92.2% vs. 12.5%) while maintaining high sensitivity (89.3% vs. 100%). Morphological correlation showed that Galectin-7 expression was restricted to Ba/Sq-differentiated tumor nests and absent in UC areas, supporting its utility in distinguishing true squamous differentiation. Importantly, Kaplan–Meier analysis revealed that Galectin-7 positivity stratified patients with worse progression-free survival, suggesting that Galectin-7 is not only a diagnostic marker but also a prognostic indicator of aggressive disease. Although the difference did not reach statistical significance in our current cohort, the survival curves showed a clear separation between Galectin-7–positive and –negative cases. This is likely due to the limited sample size and the exclusion of patients who received neoadjuvant chemotherapy or BCG therapy, which may have reduced statistical power. Notably, subgroup analyses stratified by T stage, N stage, and overall clinical stage did not demonstrate statistically significant differences, further suggesting that the prognostic effect of Galectin-7 cannot be explained solely by stage distribution, although statistical power was limited. Larger studies will be required to validate the prognostic significance of Galectin-7. This finding parallels previous reports that the loss of urothelial differentiation markers such as FOXA1 is associated with high-grade, late-stage UC and poor outcomes [46].

Hayashi et al. [22] previously identified desmocollin-2 (DSC2) as a highly sensitive and specific immunohistochemical marker for squamous differentiation in UC. However, our spatial transcriptomic data showed that DSC2 transcripts were not strictly confined to Ba/Sq-differentiated tumor nests and displayed spillover into UC regions. In contrast, Galectin-7 exhibited sharp spatial restriction to the basal-like compartment characteristic of early Ba/Sq differentiation and declined with terminal keratinization, making it a more precise molecular correlate of squamous onset. These findings, in line with previous reports on CAF-driven plasticity and the tumor microenvironment’s influence on lineage fate [24, 28], suggest that Galectin-7 integrates stromal and transcriptional signals to mark early squamous commitment. Thus, Galectin-7 appears to function as a p63-associated effector linked to early squamous differentiation rather than merely serving as a correlative marker.

This study has several limitations. First, Visium HD spatial transcriptomics was performed on only one representative case, which limits the generalizability of the findings. Larger patient cohorts are required to validate the molecular trajectory of the Ba/Sq differentiation process within UC and to confirm the consistency of NGFR and Galectin-7 as key regulatory nodes in this process. Second, functional validation of NGFR signaling and CAF plasticity was not conducted; mechanistic studies using lineage tracing or perturbation models will be necessary to establish causality [42, 43]. Third, although our immunohistochemical validation included 28 Ba/Sq UC and 64 pure UC cases, the cohort was restricted to specific clinical conditions (≥ T2 stage, no neoadjuvant chemotherapy, no BCG therapy), which may limit the broader applicability of the results. Recent advances in spatial multi-omics and computational deconvolution approaches have demonstrated the potential of high-resolution platforms to dissect complex tissue microenvironments [47, 48]. Integration of such multi-modal or single-cell–informed analyses will further strengthen the robustness of Ba/Sq differentiation trajectory mapping within UC.

Future research should incorporate high-resolution single-cell technologies, such as Xenium and other spatially resolved single-cell platforms, to achieve finer delineation of stromal–epithelial signaling axes and the mechanisms underlying squamous lineage commitment [21]. From a translational perspective, Galectin-7 represents a promising candidate not only for diagnostic application but also for therapeutic development. Antibody–drug conjugates or inhibitory agents targeting Galectin-7 could potentially offer Ba/Sq-specific treatment strategies that complement existing UC-directed regimens. Such targeted approaches may ultimately improve clinical outcomes in Ba/Sq UC, which currently lacks lineage-specific therapies.

## Conclusions

In summary, our spatial transcriptomic and immunohistochemical analyses provide a stepwise molecular framework for understanding Ba/Sq differentiation within urothelial carcinoma. We identify a stromal–epithelial signaling axis in which CAF-derived cues activate NGFR-positive basal-like cells, leading to p63-driven induction of Galectin-7 and subsequent squamous differentiation. Importantly, Galectin-7 emerged as a highly specific diagnostic marker that outperformed p63 and correlated with poor prognosis. Although this study was based on a single representative case, our findings establish Galectin-7 as both a mechanistic participant and a clinically relevant biomarker, warranting further validation in larger patient cohorts.

## Methods

### Diagnosis of UC–Ba/Sq case

We retrospectively reviewed bladder tumor specimens resected at Ehime University Hospital and identified a case of urothelial carcinoma with squamous differentiation (UC with Ba/Sq differentiation). Diagnosis was made in accordance with the 2022 WHO Classification of the Urinary System and was independently confirmed by two board-certified pathologists and one urologist. Hematoxylin–eosin (H&E) stained sections were evaluated with particular attention to the UC–Ba/Sq differentiation interface to ensure the presence of contiguous UC and Ba/Sq-differentiated components within a single tissue section suitable for spatial transcriptomic analysis. This representative case was selected because it exhibited a well-demarcated and continuous UC with Ba/Sq differentiation within one surgical specimen, allowing precise spatial mapping of molecular changes across the differentiation interface.

This study was approved by the Institutional Review Board of Ehime University (Approval No. 2207001) and conducted in accordance with the principles of the Declaration of Helsinki. An opt-out notice regarding the research use of patient samples and clinical data was posted on the institutional website. The requirement for informed consent was waived by the Clinical Research Ethics Committee, in accordance with institutional guidelines.

### Spatial transcriptomics (Visium HD)

Spatial transcriptomic analysis was performed as previously described [49], with modifications specific to the Visium HD platform. Visium HD was selected for its ability to achieve subcellular-level transcript capture, enabling high-resolution mapping of the UC–Ba/Sq interface. Formalin-fixed paraffin-embedded (FFPE) tumor sections passing RNA quality control (DV200 > 30%) were used for spatial library construction and sequencing. Sections were cut at a thickness of 10 μm and mounted on Visium HD slides. The entire capture area (6.5 × 6.5 mm²) was selected to encompass both urothelial carcinoma (UC) and Ba/Sq-differentiated tumor regions, with particular attention to the UC–Ba/Sq interface.

Tissue sections were deparaffinized, stained with H&E, and imaged before probe hybridization. Transcript capture, probe ligation, extension, and reverse transcription were performed according to the Visium HD Spatial Gene Expression for FFPE Tissue Preparation Guide (10x Genomics, CG000518). cDNA amplification and library construction followed the standard Visium HD workflow.

Sequencing was carried out using the MGI DNBSEQ-G400RS platform (MGI Tech Co., Shenzhen, China). Raw data were processed using the Space Ranger HD pipeline (10x Genomics, version spaceranger-2.1.1) with the GRCh38-2020-A reference transcriptome.

### Quality control

The sequencing output demonstrated robust performance, yielding 671,854 bins under tissue, with a mean of approximately 756 reads and 120 UMIs per 8 μm bin. In total, 18,008 genes were detected, with more than 85% of barcodes being valid and over 97% of reads confidently mapped to the probe set.

Spatial alignment of barcoded bins with tissue morphology was visually confirmed using Loupe Browser (10x Genomics). Downstream clustering, differential gene expression analysis, and data visualization (UMAPs, spatial feature plots, and violin plots) were performed in Loupe Browser.

### Data processing and normalization

Raw sequencing data from the UC with Ba/Sq differentiation specimen were processed using the Space Ranger pipeline (10x Genomics, version spaceranger-2.1.1) with default parameters. The resulting feature-barcode matrix was analyzed in Loupe Browser (10x Genomics, Pleasanton, CA, USA). Cluster annotation, differential gene expression analysis, and data visualization were conducted following previously described protocols [50, 51]. Spot-level gene expression counts were normalized using log1p(CP10k) prior to downstream analyses and visualization. This normalization approach was chosen because it corrects for library size differences and stabilizes variance across genes, providing comparable expression values between spatial spots.

Quality control metrics confirmed robust data quality, with 85.7% valid barcodes, 97.3% of reads confidently mapped to the probe set, and a median of approximately 120 genes detected per 8 μm bin. Over 99% of bins were located beneath tissue, and spatial alignment of UMIs with tissue morphology was visually verified in Loupe Browser.

### Differential gene expression and cluster annotation

Unsupervised clustering was performed in Loupe Browser using default settings. Differentially expressed genes (DEGs) for each cluster were identified relative to all other clusters based on normalized UMI counts using Loupe Browser’s built-in statistical framework. Clusters were manually annotated based on the average normalized expression of canonical marker genes. These included luminal UC markers (e.g., GATA3, UPK1A/UPK2, KRT20), basal/squamous markers (e.g., TP63, KRT5/14, DSG3), keratinization markers (e.g., IVL, SPRR family, LOR), fibroblast markers (e.g., COL1A1, DCN, FAP, ACTA2, TAGLN), and immune markers (e.g., IGKC, MS4A1, CD79A).

The complete list of annotation genes is provided in Table 1.

**Table 1:** Canonical marker genes used for cluster annotation

Cluster	Annotation	Marker genes used for annotation
1	Plasma cell–rich B cell cluster	MZB1, JCHAIN, XBP1, IGKC
2	NGFR⁺ basal-like progenitor cluster	NGFR, TP63, KRT14, ITGA6, KRT5, SOX9
3	myCAF (myofibroblastic CAF)	TAGLN, MYL9, CNN1, ACTA2
4	Luminal UC cluster	UPK1A, UPK2, KRT20, GATA3, FOXA1
5	Basal-like SCC cluster	DSG3, SFN, TP63, KRT5, EGFR
6	Keratinizing SCC cluster (high-keratinization)	LCE1A, FLG2, KLK5, KLK7, CNFN, RPTN
7	Inflammation-associated SCC cluster	KRT6A, KRT17, CXCL8, S100A8, S100A9, IL1B, NFKBIA
8	iCAF (inflammatory CAF)	FAP, POSTN, CXCL12, COL1A1, TGFB1, IL6
9	Germinal center B cell cluster	MS4A1, CD79A, POU2AF1, BANK1
10	Terminally differentiated SCC cluster	KLK7, KLK5, CASP14, LCE3D, DSG1

### Functional enrichment analysis

Cluster-specific DEGs were subjected to functional enrichment analysis using *Metascape* (https://metascape.org) to identify significantly enriched Gene Ontology (GO) terms and signaling pathways. Results were visualized as bar plots ranked by –log10(p-value). Analyses focused on comparisons between UC and Ba/Sq-differentiated clusters, as well as among fibroblast clusters, to investigate pathways involved in the Ba/Sq differentiation process within UC.

### Cluster annotation and marker gene visualization

Normalized gene expression values (log1p(CP10k)) were projected onto UMAP embeddings and spatial tissue maps. While all spots were visualized faintly, annotated clusters were highlighted based on the average expression of selected canonical marker genes (see Table 1).

### Statistical analysis

Kaplan–Meier analysis was used to estimate survival curves, and differences between groups were evaluated using the log-rank test. For in vitro proliferation assays (*n* = 3 independent experiments), statistical significance was assessed using a two-tailed Student’s *t*-test. The diagnostic performance of Galectin-7 immunohistochemistry was evaluated by calculating sensitivity and specificity. All statistical analyses were performed using EZR (version 1.68; Saitama Medical Center, Jichi Medical University, Saitama, Japan), which is a graphical user interface for R software (The R Foundation for Statistical Computing, Vienna, Austria). A *p*-value < 0.05 was considered statistically significant.

### Cell culture

The SCaBER cell line (ATCC HTB-3), derived from the urinary bladder of a 58-year-old Black male patient with pure squamous cell carcinoma, was used in this study. Cells were obtained from the American Type Culture Collection (ATCC, Manassas, VA, USA) and cultured under standard conditions as previously described [52]. Cells were maintained in MEM supplemented with 10% fetal bovine serum (FBS), 100 U/mL penicillin, and 100 µg/mL streptomycin at 37 °C in a humidified incubator with 5% CO₂. Cells were used between passages 5–20 for all experiments. Cell line authentication was performed by short tandem repeat (STR) profiling, and all cultures were confirmed to be free of mycoplasma contamination.

### Western blotting

Western blotting was performed as previously described [43]. Briefly, cells were lysed in RIPA buffer supplemented with protease and phosphatase inhibitors, and protein concentrations were determined using the BCA assay. Equal amounts of protein lysates were resolved by SDS-PAGE and transferred to PVDF membranes. After blocking with 5% skim milk in TBS-T, membranes were incubated with the indicated primary antibodies, followed by HRP-conjugated secondary antibodies. Signals were detected using enhanced chemiluminescence reagents.

### Immunofluorescence (IF)

Immunofluorescence staining was performed essentially as previously reported [42]. Cells grown on coverslips were fixed with 4% paraformaldehyde and permeabilized with 0.1% Triton X-100 in PBS. After blocking with 3% bovine serum albumin, cells were incubated with the indicated primary antibodies, followed by fluorescent dye–conjugated secondary antibodies. Nuclei were counterstained with Hoechst 33,342. Images were acquired using a confocal laser scanning microscope and analyzed with ImageJ software.

### Antibodies

The following antibodies were used in this study:

For immunohistochemistry (IHC), rabbit anti-Galectin-7 antibody (EPR4287, ab108623, dilution 1:1500; Abcam), mouse anti-p63 antibody (clone 4A4, ready-to-use; Nichirei Biosciences), and goat normal serum for blocking (Vector Laboratories) were used. For Western blotting and immunofluorescence (WB/IF), rabbit anti-Galectin-7 (ab10482, dilution 1:1000; Abcam) was used.

For secondary detection in IHC, the Histofine Simple Stain MAX-PO (Nichirei Biosciences) was applied without dilution. Antibody dilutions were prepared using Antibody Diluent (S0809; Dako). Nuclear counterstaining was performed using hematoxylin, and signals were visualized using DAB (3,3′-diaminobenzidine; Nichirei Biosciences).

### Immunohistochemistry

IHC was performed according to standard protocols [53], with minor modifications. Briefly, 4 μm sections from formalin-fixed, paraffin-embedded (FFPE) tissue blocks were deparaffinized in xylene and rehydrated through a graded ethanol series. Antigen retrieval was conducted by microwave heating in citrate buffer (pH 6.0). Endogenous peroxidase activity was quenched with 3% hydrogen peroxide for 10 min at room temperature.

Sections were then incubated overnight at 4 °C with primary antibodies against p63 (mouse monoclonal, clone 4A4, ready-to-use; Nichirei Biosciences) and Galectin-7 (rabbit monoclonal, clone EPR4287, 1:1500; Abcam). Detection was performed using the Histofine Simple Stain MAX-PO system and visualized with DAB. Slides were counterstained with hematoxylin, dehydrated, and mounted with coverslips. Negative controls were prepared by omitting the primary antibody.

Galectin-7 immunohistochemical positivity was independently assessed by two urologists and one board-certified pathologist, all blinded to clinical outcomes. Cases were classified as positive or negative based on overall staining intensity and distribution. In cases of discrepant assessment, slides were reviewed jointly and a consensus diagnosis was reached.

## Supplementary Information

Below is the link to the electronic supplementary material.

Supplementary Material 1. Figure 1. Transcriptomic features defining individual clusters. (A) Heatmap showing the top 20 upregulated genes defining each of the ten clusters. (B) Pathway enrichment analysis of differentially expressed genes in each cluster. Figure S2. Detailed clinicopathological characteristics of Galectin-7–positive and –negative urothelial carcinoma cases. Comprehensive summary of clinicopathological features according to Galectin-7 (G7) expression status, including patient age, squamous differentiation (Ba/Sq), p63 expression, sex, pathological T and N stages, overall stage, grade, and surgical procedure. Median progression-free survival (mPFS) and overall survival (mOS) are also shown. Figure S3. Subgroup survival analyses stratified by clinicopathological stage. Subgroup survival analyses stratified by pathological T stage, N stage, and overall clinical stage. Kaplan–Meier curves for progression-free survival (PFS) and overall survival (OS) according to Galectin-7 expression are shown for each subgroup. Hazard ratios (HRs) with 95% confidence intervals were calculated using Cox proportional hazards models.

## Data Availability

The data supporting the findings of this study are available from the corresponding author upon reasonable request. Raw and processed spatial transcriptomic data generated in this study are openly available in the Gene Expression Omnibus (GEO) under accession number GSE299573 (https://www.ncbi.nlm.nih.gov/geo/).The associated raw FASTQ files are also available in the NCBI Sequence Read Archive (SRA), an INSDC member repository, under the following accessions: BioProject PRJNA1274999, BioSample SAMN49010684, and SRA SRX29144058.
