# Supplementary material for: Spatial transcriptomics of urothelial carcinoma with basal/squamous differentiation identifies Galectin-7 as a specific marker of squamous lineage commitment
Source: Discov Oncol. 2026 Mar 31;17:722. doi: 10.1007/s12672-026-04927-z (PMC13168405; doi:10.1007/s12672-026-04927-z)
Supplement: Supplementary file 1 — Supplementary Material 1. Figure 1. Transcriptomic features defining individual clusters. (A) Heatmap showing the top 20 upregulated genes defining each of the ten clusters. (B) Pathway enrichment analysis of differentially expressed genes in each cluster. Figure S2. Detailed clinicopathological characteristics of Galectin-7–positive and –negative urothelial carcinoma cases. Comprehensive summary of clinicopathological features according to Galectin-7 (G7) expression status, including patient age, squamous differentiation (Ba/Sq), p63 expression, sex, pathological T and N stages, overall stage, grade, and surgical procedure. Median progression-free survival (mPFS) and overall survival (mOS) are also shown. Figure S3. Subgroup survival analyses stratified by clinicopathological stage. Subgroup survival analyses stratified by pathological T stage, N stage, and overall clinical stage. Kaplan–Meier curves for progression-free survival (PFS) and overall survival (OS) according to Galectin-7 expression are shown for each subgroup. Hazard ratios (HRs) with 95% confidence intervals were calculated using Cox proportional hazards models. [file 12672_2026_4927_MOESM1_ESM.docx]

**Supplementary Figure 1. Transcriptomic features defining individual clusters.**
(A) Heatmap showing the top 20 upregulated genes defining each of the ten clusters.
(B) Pathway enrichment analysis of differentially expressed genes in each cluster.

**Supplementary Figure 2. Detailed clinicopathological characteristics of Galectin-7–positive and –negative urothelial carcinoma cases.**

Comprehensive summary of clinicopathological features according to Galectin-7 (G7) expression status, including patient age, squamous differentiation (Ba/Sq), p63 expression, sex, pathological T and N stages, overall stage, grade, and surgical procedure.
Median progression-free survival (mPFS) and overall survival (mOS) are also shown.

**Supplementary Figure 3. Subgroup survival analyses stratified by clinicopathological stage.**

Subgroup survival analyses stratified by pathological T stage, N stage, and overall clinical stage. Kaplan–Meier curves for progression-free survival (PFS) and overall survival (OS) according to Galectin-7 expression are shown for each subgroup. Hazard ratios (HRs) with 95% confidence intervals were calculated using Cox proportional hazards models.


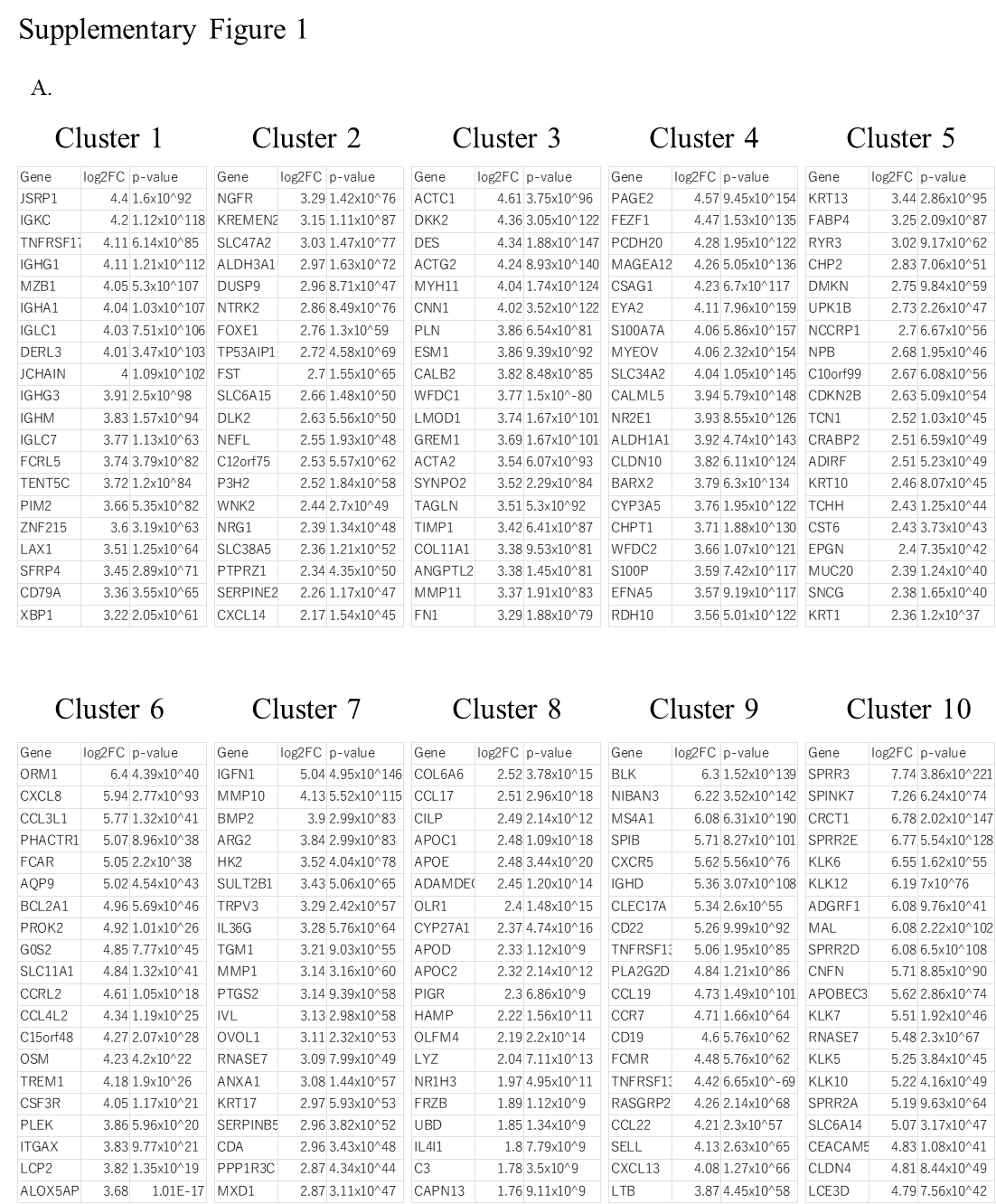


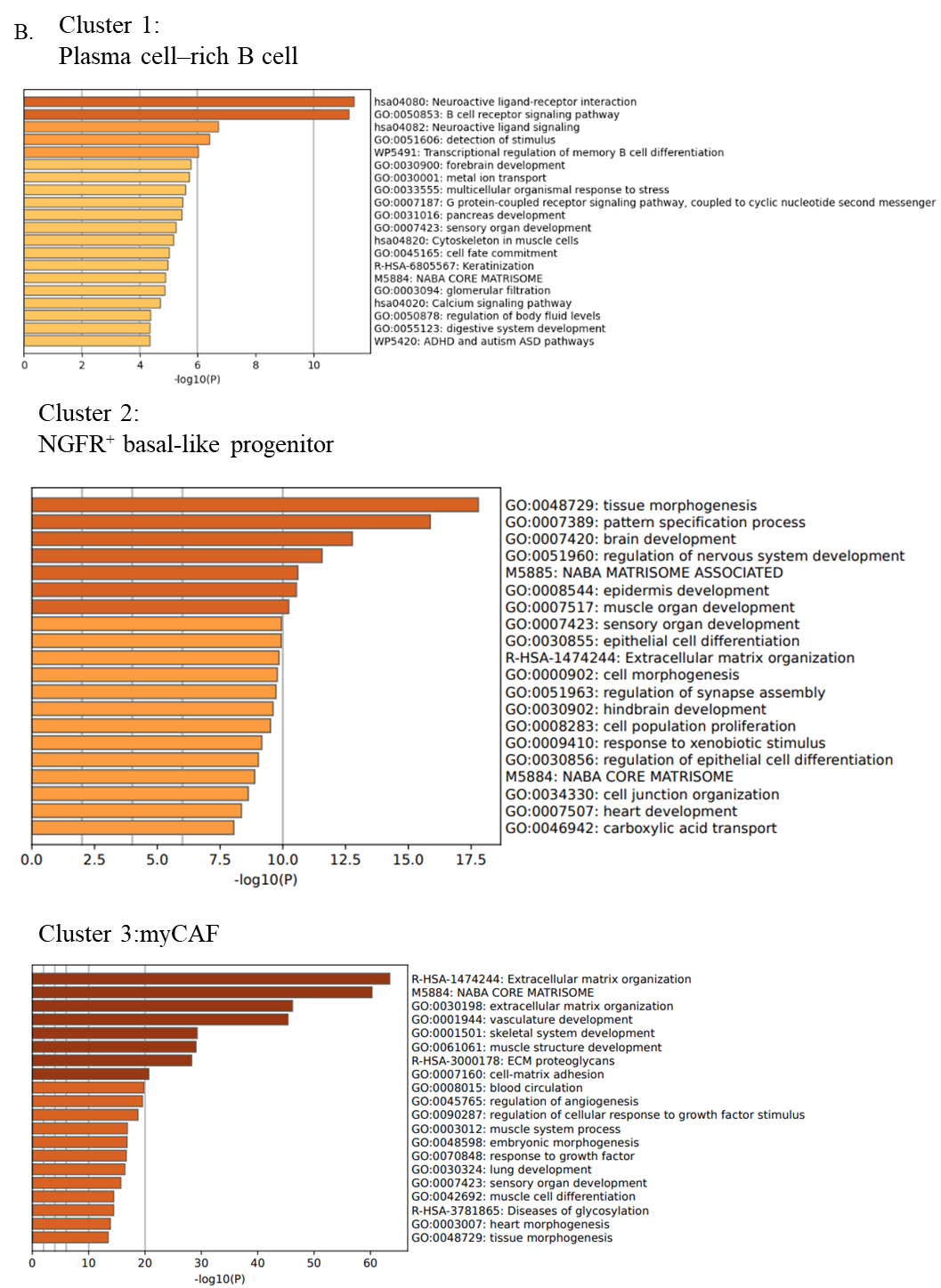


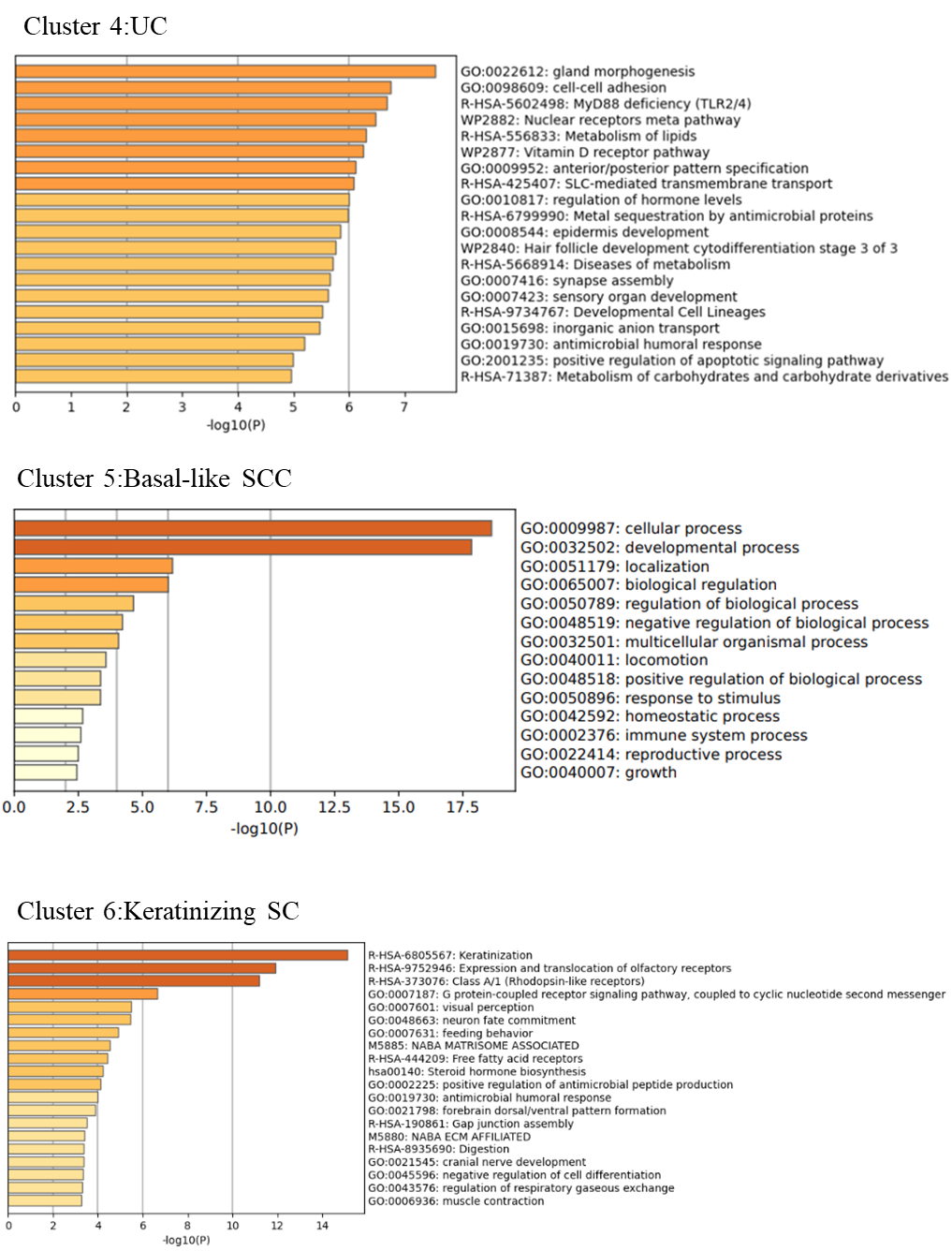


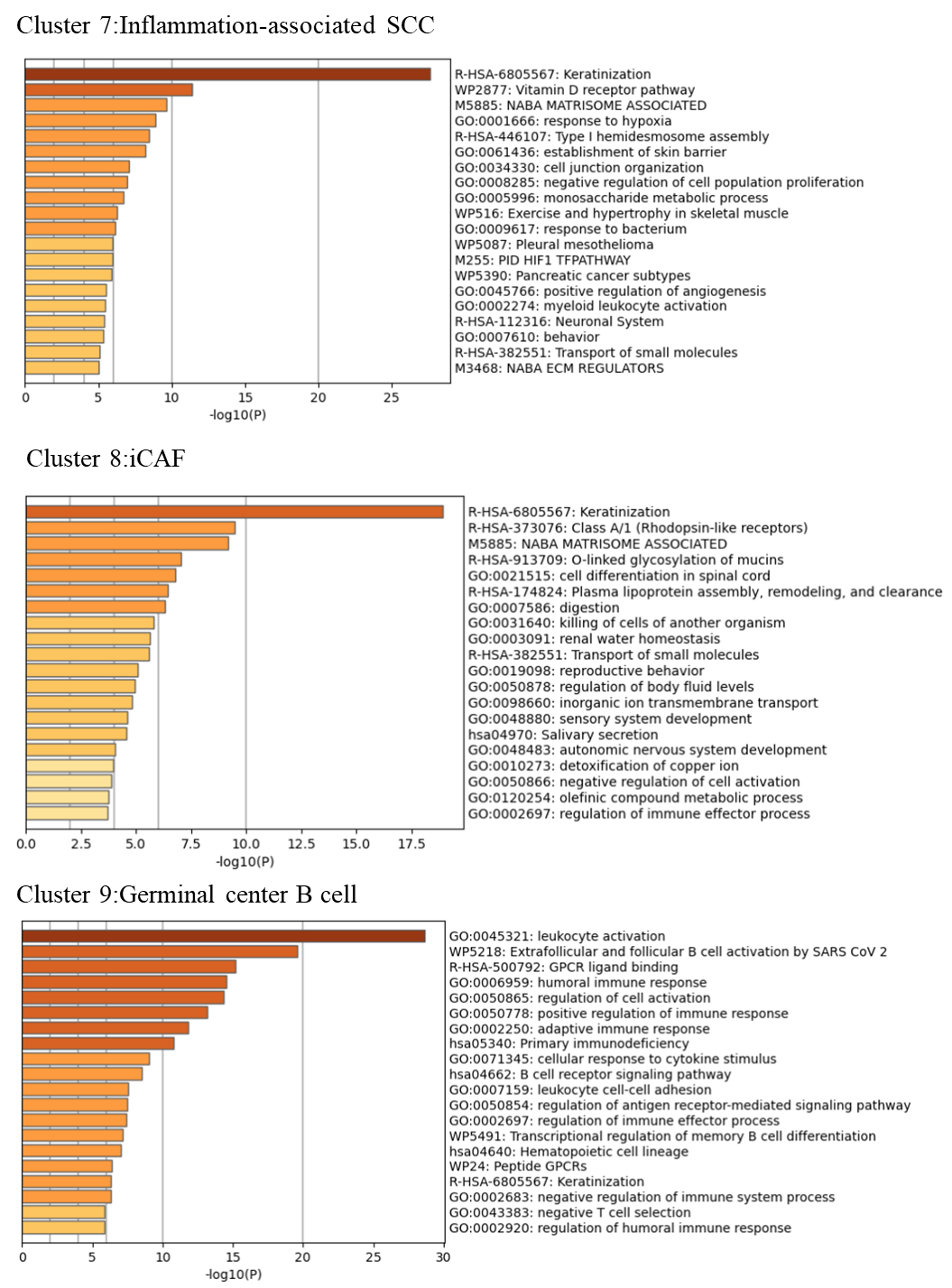


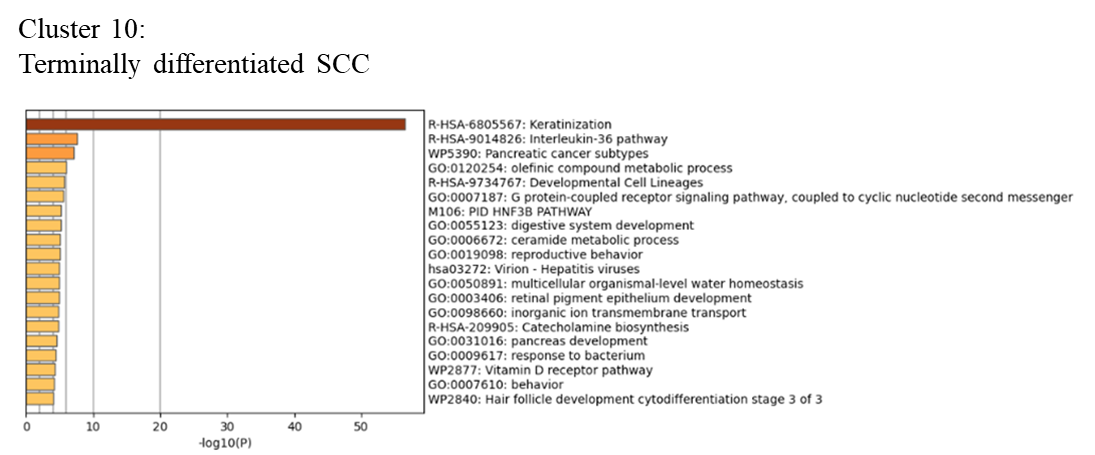


Supplementary Figure 2

|  |  | G7 positive | G7 negative |
| --- | --- | --- | --- |
|  |  | n=14 | n=41 |
| mAge |  | 78.0 (53-93) | 77.0 (59-91) |
| BaSq | positive | 10 (71.4%) | 2 (4.9%) |
|  | negative | 4 (28.6%) | 39 (95.1%) |
| mPFS |  | 258.0 (21-2519) | 463.0 (17-2754) |
| mOS |  | 498.5 (21-2519) | 885.0 (17-2978) |
| p63 | positive | 14 (100.0%) | 39 (95.1%) |
|  | negative | 0 (0.0%) | 2 (4.9%) |
| Sex | Male | 7 (50.0%) | 36 (95.1%) |
|  | Female | 7 (50.0%) | 5 (12.2%) |
| pT | 0 | 1 (7.1%) | 19 (46.3%) |
|  | 1 | 3 (21.4%) | 4 (9.8%) |
|  | 2 | 3 (21.4%) | 8 (19.5%) |
|  | 3 | 6 (42.9%) | 4 (9.8%) |
|  | 4 | 1 (7.1%) | 6 (14.6%) |
| pN | 0 | 12 (85.7%) | 36 (87.8%) |
|  | 1 | 2 (14.3%) | 2 (4.9%) |
|  | 2 | 0 (0.0%) | 3 (7.3%) |
| Stage | 1 | 4 (28.6%) | 23 (56.1%) |
|  | 2 | 3 (21.4%) | 6 (14.6%) |
|  | 3 | 5 (35.7%) | 11 (26.8%) |
|  | 4 | 2 (14.3%) | 1 (2.4%) |
| Grade | 1 | 1 (7.1%) | 11 (26.8%) |
|  | 2 | 0 (0.0%) | 5 (12.2%) |
|  | 3 | 13 (92.9%) | 25 (61.0%) |
| Operation | TUR-Bt | 8 (57.1%) | 29 (61.0%) |
|  | Cystectomy | 5 (35.7%) | 9 (22.0%) |
|  | Nephrectomy | 1 (7.1%) | 3 (7.3%) |

**Supplementary Figure 3**

**
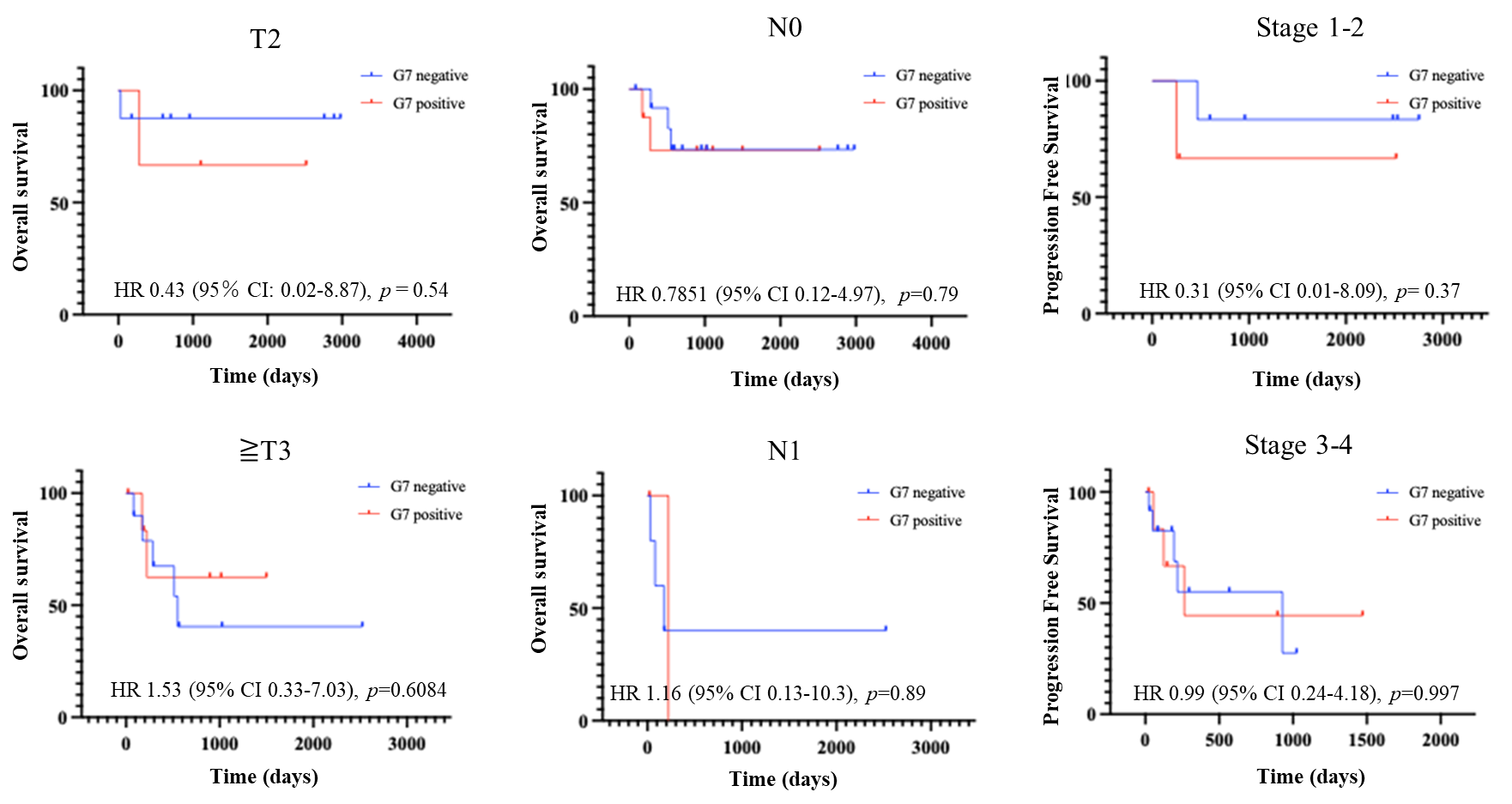
**

**
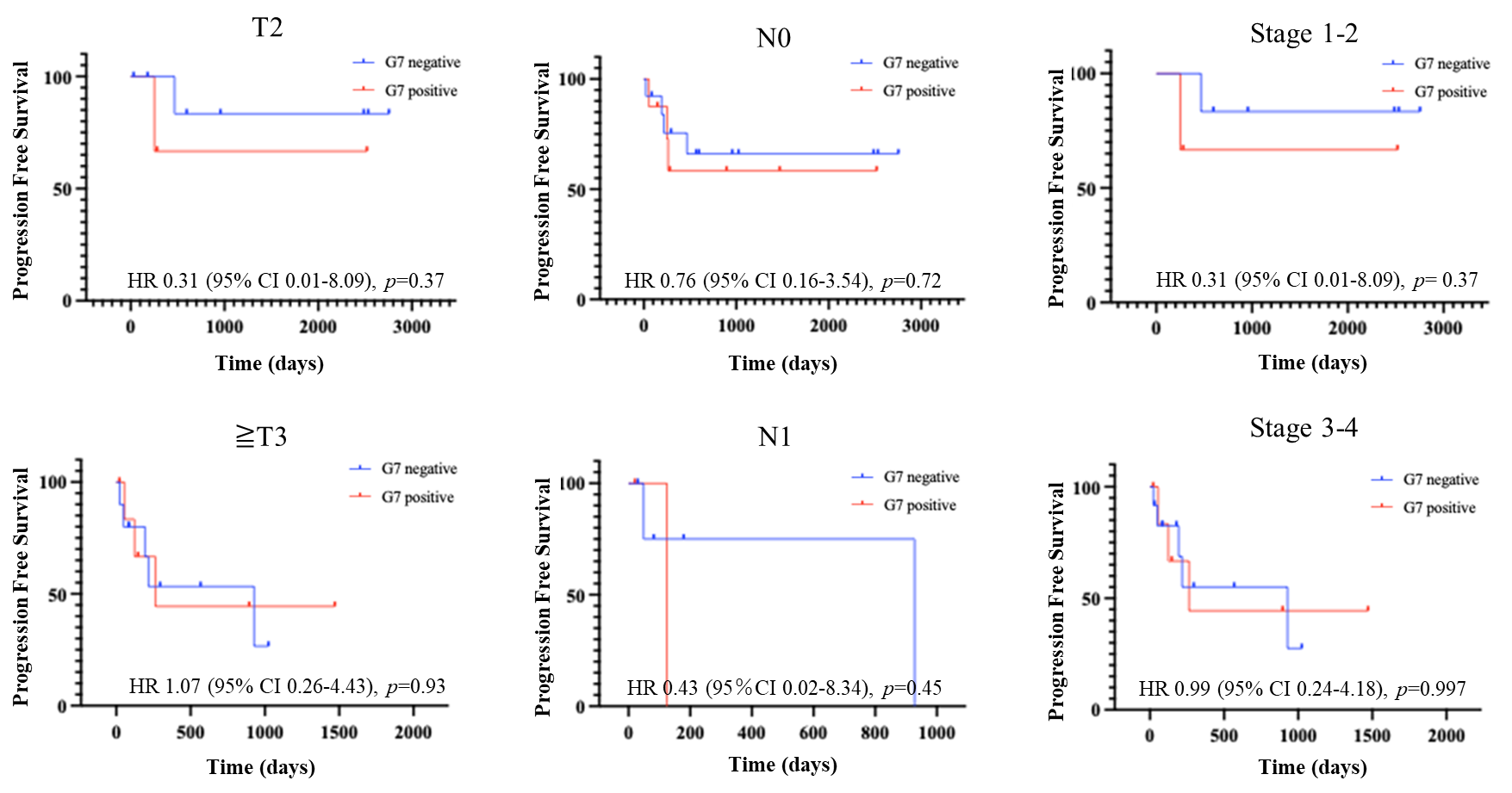
**
